# Safety, efficacy, and pharmacokinetics of gremubamab (MEDI3902), an anti-*Pseudomonas aeruginosa* bispecific human monoclonal antibody, in *P. aeruginosa*-colonised, mechanically ventilated intensive care unit patients: a randomised controlled trial

**DOI:** 10.1186/s13054-022-04204-9

**Published:** 2022-11-15

**Authors:** Jean Chastre, Bruno François, Marc Bourgeois, Apostolos Komnos, Ricard Ferrer, Galia Rahav, Nicolas De Schryver, Alain Lepape, Iftihar Koksal, Charles-Edouard Luyt, Miguel Sánchez-García, Antoni Torres, Philippe Eggimann, Despoina Koulenti, Thomas L. Holland, Omar Ali, Kathryn Shoemaker, Pin Ren, Julien Sauser, Alexey Ruzin, David E. Tabor, Ahmad Akhgar, Yuling Wu, Yu Jiang, Antonio DiGiandomenico, Susan Colbert, Drieke Vandamme, Frank Coenjaerts, Surbhi Malhotra-Kumar, Leen Timbermont, Antonio Oliver, Olivier Barraud, Terramika Bellamy, Marc Bonten, Herman Goossens, Colin Reisner, Mark T. Esser, Hasan S. Jafri, The COMBACTE-MAGNET EVADE Study Group, Michael Joannidis, Walter Klimscha, Elisabeth De Waele, Jacques Devriendt, Vincent Huberlant, Pieter Depuydt, Sam Van Boxstael, Mladen Peric, Jasminka Kopic, Michal Hanauer, Tomas Hruby, Vladimir Sramek, Petr Svoboda, Tomas Vymazal, Martin Novacek, Djillali Annane, Jean-Paul Mira, Bertrand Souweine, Pierre-François Dequin, Ferhat Meziani, François Stephan, Saadalla Nseir, Sebastien Gibot, Carole Schwebel, Gaetan Plantefeve, Jean-Luc Diehl, Christian Richard, Christian Lamer, Kada Klouche, Samir Jaber, Epaminondas Zakynthinos, Georgios Filntisis, Spyros Zakynthinos, Antonia Koutsoukou, Georgios Saroglou, Charikleia Nikolaou, Glykeria Vlachogianni, Ioannis Pnevmatikos, Konstantinos Mandragos, Ildiko Kremer, Zsolt Dezso Rozgonyi, Zsuzsa Marjanek, Ignacio Martin-Loeches, Pierre Singer, Vernon Van Heerden, Yehuda Carmeli, Pedro Povoa, Antonio Alvarez Seoane, Pedro Moura, Filipe Gonzalez, Paula Ramirez, Antonio Torres Marti, Ricard Ferrer Roca, Lorena Oteiza, Dolores Escudero, Enrique Piacentini, Paula Vera, Luis Tamayo, Miguel Angel Gonzalez Gallego, Borja Suberviola Canas, Iglesias Figueira, Rafael Leon, Volkan Korten, Murat Akova, Duncan Wyncoll, Tony Whitehouse, Phil Hopkins, Malcolm Sim, Yoav Golan, Marcus Zervos, Jose Vazquez, Kartikeya Cherabuddi, George Smulian, Nadine Rouphael, James Welker, Mathew Sims, David Van Duin, Todd McCarthy, Christopher Polk

**Affiliations:** 1 Service de Médecine Intensive Réanimation, Institut de Cardiologie, Groupe Hospitalier Pitié-Salpêtrière, Assistance Publique-Hôpitaux de Paris, Sorbonne University, 47–83 Bd de l’Hôpital, 75651 Paris, France; 2 Réanimation Polyvalente and Inserm CIC 1435 & UMR 1092, CHU, Limoges, France; 3 AZ Sint-Jan Brugge-Oostende AV, Brugge, Belgium; 4 General Hospital of Larissa, Larissa, Greece; 5 SODIR-VHIR Research Group, Hospital Universitari Vall d’Hebron, Barcelona, Spain; 6 Chaim Sheba Medical Center, Sackler School of Medicine, Tel Aviv University, Tel Aviv, Israel; 7 Clinique Saint-Pierre, Ottignies, Belgium; 8 Hospices Civils de Lyon Hôpital Lyon Sud, Lyon, France; 9 Faculty of Medicine, Trabzon and Acibadem University Faculty of Medicine, Karadeniz Technical University, Istanbul, Turkey; 10 Critical Care Department, Hospital Clínico San Carlos, Universidad Complutense, Madrid, Spain; 11 Servei de Pneumologia, Hospital Clinic, University of Barcelona, IDIBAPS, CIBERES, ICREA, Barcelona, Spain; 12 Department of Locomotor Apparatus, Centre Hospitalier Universitaire Vaudois CHUV, Lausanne, Switzerland; 13 The University of Queensland Centre for Clinical Research, Faculty of Medicine, The University of Queensland, Brisbane, QLD Australia; 14 2nd Critical Care Department, Attikon University Hospital, National and Kapodistrian, University of Athens, Athens, Greece; 15 Duke Clinical Research Institute, Durham, NC USA; 16 Clinical Research and Development, Vaccines and Immune Therapies, BioPharmaceuticals R&D, AstraZeneca Biopharmaceuticals, One MedImmune Way, Gaithersburg, MD 20878 USA; 17 Respiratory and Immunology, BioPharmaceuticals R&D, AstraZeneca, Gaithersburg, MD USA; 18 Infection Control Program, Faculty of Medicine, Geneva University Hospitals, Geneva, Switzerland; 19 Clinical Pharmacology and Quantitative Pharmacology, Clinical Pharmacology and Safety Sciences, BioPharmaceuticals R&D, AstraZeneca, Gaithersburg, MD USA; 20 Oncology, US SM&M, AstraZeneca, Wilmington, NC USA; 21 Inserm CIC 1435, CHU, Limoges, France; 22 Department of Medical Microbiology, University Medical Center Utrecht, Utrecht University, Utrecht, The Netherlands; 23 Laboratory of Medical Microbiology, Vaccine and Infectious Disease Institute, University of Antwerp, Antwerp, Belgium; 24 Servicio de Microbiología y Unidad de Investigación, Hospital Universitari Son Espases, Institut d’Investigació Sanitaria Illes Balears, Palma, Spain; 25 INSERM U1092, Centre Hospitalier Universitaire de Limoges, Université Limoges, Limoges, France; 26 Julius Center for Health Science and Primary Care, University Medical Center Utrecht, Utrecht University, Utrecht, The Netherlands; 27 DevPro Biopharma, Basking Ridge, NJ USA; 28 Late-Stage Development, Respiratory and Immunology, BioPharmaceuticals R&D, AstraZeneca, Gaithersburg, MD USA

**Keywords:** Monoclonal antibody, Prevention, Pharmacokinetics, *Pseudomonas aeruginosa* ventilator-associated pneumonia, Safety

## Abstract

**Background:**

Ventilator-associated pneumonia caused by *Pseudomonas aeruginosa* (PA) in hospitalised patients is associated with high mortality. The effectiveness of the bivalent, bispecific mAb MEDI3902 (gremubamab) in preventing PA nosocomial pneumonia was assessed in PA-colonised mechanically ventilated subjects.

**Methods:**

EVADE (NCT02696902) was a phase 2, randomised, parallel-group, double-blind, placebo-controlled study in Europe, Turkey, Israel, and the USA. Subjects ≥ 18 years old, mechanically ventilated, tracheally colonised with PA, and without new-onset pneumonia, were randomised (1:1:1) to MEDI3902 500, 1500 mg (single intravenous dose), or placebo. The primary efficacy endpoint was the incidence of nosocomial PA pneumonia through 21 days post-dose in MEDI3902 1500 mg versus placebo, determined by an independent adjudication committee.

**Results:**

Even if the initial sample size was not reached because of low recruitment, 188 subjects were randomised (MEDI3902 500/1500 mg: *n* = 16/87; placebo: *n* = 85) between 13 April 2016 and 17 October 2019. Out of these, 184 were dosed (MEDI3902 500/1500 mg: *n* = 16/85; placebo: *n* = 83), comprising the modified intent-to-treat set. Enrolment in the 500 mg arm was discontinued due to pharmacokinetic data demonstrating low MEDI3902 serum concentrations. Subsequently, enrolled subjects were randomised (1:1) to MEDI3902 1500 mg or placebo. PA pneumonia was confirmed in 22.4% (*n* = 19/85) of MEDI3902 1500 mg recipients and in 18.1% (*n* = 15/83) of placebo recipients (relative risk reduction [RRR]: − 23.7%; 80% confidence interval [CI] − 83.8%, 16.8%; *p* = 0.49). At 21 days post-1500 mg dose, the mean (standard deviation) serum MEDI3902 concentration was 9.46 (7.91) μg/mL, with 80.6% (*n* = 58/72) subjects achieving concentrations > 1.7 μg/mL, a level associated with improved outcome in animal models. Treatment-emergent adverse event incidence was similar between groups.

**Conclusions:**

The bivalent, bispecific monoclonal antibody MEDI3902 (gremubamab) did not reduce PA nosocomial pneumonia incidence in PA-colonised mechanically ventilated subjects.

*Trial registration* Registered on Clinicaltrials.gov (NCT02696902) on 11th February 2016 and on EudraCT (2015-001706-34) on 7th March 2016.

**Supplementary Information:**

The online version contains supplementary material available at 10.1186/s13054-022-04204-9.

## Introduction

*Pseudomonas aeruginosa* (PA) is a common cause of ventilator-associated pneumonia (VAP) in hospitalised patients [1–3], associated with mortality rates > 30% in patients with antibiotic-susceptible and > 44% with multidrug-resistant (MDR) strains [4, 5]. Tracheobronchial colonisation with PA increases the odds of developing VAP by around eightfold [2, 6], but no specific guidelines exist for managing these patients before pneumonia is diagnosed. Current strategies involve antibiotic treatment post-diagnosis [1, 7, 8]. Although evidence suggests the earlier treatment may be beneficial [9], routine prophylactic antibiotics to prevent VAP can contribute to PA resistance [8, 10, 11]. Consequently, Infectious Diseases Society of America and the American Thoracic Society guidelines recommend withholding antibiotic treatment in patients with suspected VAP and endotracheal culture results below the diagnostic threshold for VAP (protected specimen brush [PSB] with < 10^3^ colony-forming units [CFU]/mL, bronchoalveolar lavage [BAL] with < 10^4^ CFU/mL) [12]. No systemic agents are currently approved for the pre-emptive treatment of ventilated patients with *Pseudomonas* airway colonisation to prevent PA pneumonia [7, 13], highlighting an unmet need for effective, targeted prevention.

Monoclonal antibodies are an attractive alternative to systemic antibiotics for the pre-emption of PA pneumonias. Their benefits include enhanced specificity, longer half-life, and complementary mechanism of action to antibiotics and do not induce antibiotic resistance [7, 14]. A placebo-controlled phase 2a study highlighted the potential for reducing PA pneumonia incidence in colonised, mechanically ventilated intensive care unit (ICU) patients dosed with a monovalent, monoclonal antibody against the PA PcrV protein [6].

MEDI3902 (gremubamab) is a first-in-class bivalent, bispecific human immunoglobulin G1 kappa monoclonal antibody that selectively binds to the PA PcrV protein and Psl exopolysaccharide involved in host cell cytotoxicity and PA colonisation and tissue adherence, respectively [15–17]. Prophylactic MEDI3902 administration protected against lethal PA in animal models [15, 18], with a significant reduction in the expression of genes encoding key inflammatory cytokines in animals who received MEDI3902 versus control immunoglobulin G [18].

The use of rapid diagnostic techniques, such as real-time PCR, in ICU settings can enable prompt identification of patients with bacterial colonisation of the lower respiratory tract before the onset of nosocomial pneumonia, bypassing the time required to obtain the results of conventional microbiological cultures which can take 48–72 h. Accordingly, rapid identification of patients with respiratory PA colonisation could assist in the timely initiation of pre-emptive or curative therapies. A phase 1, placebo-controlled, dose escalation study (NCT02255760) assessed an intravenous (IV) infusion of MEDI3902 in 56 healthy adults [16]. MEDI3902 serum concentrations through day 29 remained above the target therapeutic concentration of 5.3 μg/mL (based on a murine model of PA pneumonia where mice were inoculated with PA 5 × LD100, data on file) in subjects who received the highest doses (750, 1500, and 3000 mg), and dose-dependent increases in serum anticytotoxic and opsonophagocytic killing activities were observed [16]. MEDI3902 was well tolerated, supporting further assessment in PA-colonised subjects at risk for developing PA pneumonia. However, previous studies done in ICU patients for assessing the potential usefulness of monoclonal antibodies in preventing bacterial infections, while showing some non-statistically significant trends in favour of antibodies in post hoc analyses, were based on a limited number of patients and mostly negative, rendering difficult any conclusions [6, 14, 19]. Many factors that are not influenced by monoclonal antibodies can also contribute to the development of pneumonia, including disease severity, underlying immune function and concomitant therapies, warranting further studies before concluding that MEDI3902 represents a valuable complement to conventional measures for preventing lower respiratory tract infections caused by *PA*. Here, we present the results of a single-dose, proof-of-concept study of MEDI3902 for the pre-emptive treatment of PA nosocomial pneumonia in PA-colonised, mechanically ventilated subjects in the ICU.

## Methods

### Study design

EVADE (Clinicaltrials.gov NCT02696902; EudraCT 2015-001706-34) was a phase 2, randomised, parallel-group, double-blind, placebo-controlled study of MEDI3902 in mechanically ventilated patients with PA lower respiratory tract colonisation, confirmed by a polymerase chain reaction (PCR)-based test on tracheal aspirates collected no more than 36 h before randomisation. All randomised subjects were positive for PA by PCR, while 154/184 (83.7%) had positive cultures, highlighting the greater sensitivity of PCR for detecting PA airway colonisation in ventilated patients. EVADE was performed across 48 sites in 13 countries (Europe, Turkey, the USA, and Israel; Additional file 1: Tables S1 and S2).

The study was conducted within the European public–private partnership Combatting Bacterial Resistance In Europe—Molecules Against Gram-Negative Infections (COMBACTE-MAGNET) consortium [7] in accordance with the ethical principles of the Declaration of Helsinki and the International Council for Harmonization Guidance for Good Clinical Practice. The Antibiotic Resistance Leadership group also participated. Study-related documents were reviewed and approved by the local independent ethics committees or institutional review boards. All subjects/legally acceptable representatives provided written informed consent.

### Subjects

Adults ≥ 18 years of age were eligible if they met the following key inclusion criteria: currently intubated and mechanically ventilated and expected to remain so for at least 72 h; tracheal PA colonisation as assessed by PCR (GeneXpert System with PA Xpert test cartridge [research use only], Cepheid, Sunnyvale, CA, USA) no more than 36 h and no diagnosis of new-onset pneumonia within 72 h before randomisation (patients with evidence of resolved pneumonia were eligible for inclusion); expected to survive for > 2 weeks and participate in the study through 49 days post-dose.

Key exclusion criteria were: acute confirmed or suspected pseudomonal disease or active pulmonary disease; a Clinical Pulmonary Infection Score (CPIS) of at least 6 measured no more than 24 h before treatment; an Acute Physiology and Chronic Health Evaluation-II (APACHE-II) score of at least 25 or a Sequential Organ Failure Assessment (SOFA) score of at least 12; and systemic or aerosolised colistin received for > 72 h within 96 h before randomisation. The Additional file 1: Methods detail full eligibility criteria.

The modalities of the screening process were left to the discretion of the investigators. In a minority of centres, mechanically ventilated patients were routinely screened for PA colonisation using conventional microbiological cultures of endotracheal aspirates (ETA) once or twice a week until death or weaning from mechanical ventilation, according to standard practice. When cultures grew positive for PA, informed consent was obtained and tracheal colonisation was then confirmed using PCR. The other centres did not use routine serial microbiological cultures for screening. Patients’ eligibility for study enrolment was regularly checked as long as they were expected to remain on mechanical ventilation for at least 72 h and did not meet any exclusion criteria (see above), and informed consent was obtained for using PCR on ETA.

Routine use of VAP prevention bundles was highly recommended. To check whether these prevention bundles had been implemented correctly, sites were asked to report the VAP bundle application in the electronic case report form for each enrolled patient (Table 1 and Additional file 1: Table S3).

**Table 1:** Demographics and baseline characteristics (mITT)

	MEDI3902 500 mg (*N* = 16)	MEDI3902 1500 mg (*N* = 85)	Placebo (*N* = 83)	Total (*N* = 184)
Age, years; mean (SD)	62.7 (9.3)	60.3 (15.2)	64.1 (12.9)	62.2 (13.8)
Age < 65 years; *n* (%)	7 (43.8)	42 (49.4)	39 (47.0)	88 (47.8)
Sex, male; *n* (%)	10 (62.5)	54 (63.5)	62 (74.7)	126 (68.5)
Race, *n* (%)				
Asian	0 (0.0)	0 (0.0)	1 (1.2)	1 (0.5)
Black or African American	0 (0.0)	2 (2.4)	4 (4.8)	6 (3.3)
Native Hawaiian or Other Pacific Islander	0 (0.0)	0 (0.0)	1 (1.2)	1 (0.5)
White	16 (100.0)	81 (95.3)	75 (90.4)	172 (93.5)
Other	0 (0.0)	2 (2.4)	2 (2.4)	4 (2.2)
Weight, kg; mean (SD)	82.5 (25.2)	78.8 (19.5)	84.4 (21.0)	81.6 (20.7)
Height, cm; mean (SD)	167.9 (10.0)	169.1 (9.6)	171.1 (10.0)	169.9 (9.8)
BMI, kg/m2; mean (SD)	29.5 (9.4)	27.5 (6.4)	29.0 (7.7)	28.4 (7.3)
BMI ≤ 30 kg/m^2^; *n* (%)	11 (68.8)	60 (70.6)	54 (65.1)	125 (67.9)
Clinical severity scores at baseline; mean (SD)				
APACHE-II	16.9 (2.9)*	15.3 (5.4)^†^	15.5 (5.2)^‡^	15.5 (5.1)^§^
SOFA	4.5 (2.4)*	4.4 (2.7)^†^	4.0 (2.1)	4.2 (2.4)^¶^
CPIS	3.5 (1.5)*	3.0 (1.5)^#^	3.2 (1.5)^#^	3.1 (1.5)**
Duration of mechanical ventilation; days, mean (SD)	19.5 (15.5)	25.2 (27.6)	31.1 (28.4)^††^	–
Previous PA infections ≤ 3 months before randomisation; n (%)				
Yes	7 (43.8)	25 (29.4)	31 (37.8)	63 (34.4)
No	9 (56.3)	60 (70.6)	51 (62.2)	120 (65.6)
Missing	0	0	1	1
Use of antibiotics in the 3 months before randomisation; n (%)				
Yes	14 (93.3)	59 (74.7)	63 (81.8)	136 (79.5)
No	1 (6.7)	20 (25.3)	14 (18.2)	35 (20.5)
Missing	1	6	6	13
CLSI susceptibility at baseline;^‡‡^ *n* (%)				
Any culture result, *n*	16	83	82	181
* P. aeruginosa* positive	15 (93.8)	72 (86.7)	67 (81.7)	154 (85.1)
Non-MDR	6 (37.5)	37 (44.6)	25 (30.5)	68 (37.6)
MDR^§§^	4 (25.0)	12 (14.5)	16 (19.5)	32 (17.7)
XDR^§§^	4 (25.0)	14 (16.9)	21 (25.6)	39 (21.5)
PDR^§§^	0 (0.0)	0 (0.0)	0 (0.0)	0 (0.0)
Unknown^¶¶^	1 (6.3)	9 (10.8)	5 (6.1)	15 (8.3)
* P. aeruginosa* negative^##^	1 (6.3)	11 (13.3)	15 (18.3)	27 (14.9)

	MEDI3902 500 mg (*N* = 16)	MEDI3902 1500 mg (*N* = 85)	Placebo (*N* = 83)	MEDI3902 total (*N* = 101)
*P. aeruginosa* PCR CT value; mean (SD)	28.3 (3.7)	28.5 (5.7)	29.2 (6.2)	28.5 (5.4)
White blood cell count, 10^3^/μL; mean (SD)	11.6 (4.4)	13.2 (6.3)	11.4 (6.1)	12.9 (6.1)
Absolute Neutrophil count, 10^3^/μL; mean (SD)	9.1 (4.1)	10.6 (6.0)***	8.4 (4.3)^†††^	10.4 (5.7)^‡‡‡^
Procalcitonin, μg/L; mean (SD)	4.8 (15.5)^§§§^	1.0 (2.4)^¶¶¶^	0.61 (1.3)^###^	1.5 (6.1)****
CRP, mg/dL; mean (SD)	7.9 (6.3)	14.7 (37.9)^††††^	15.1 (34.3)^¶¶¶^	13.5 (34.6)^‡‡‡‡^
Ventilator-associated pneumonia prevention^†^				
All 5 measures used, n (%)	8 (50)	36 (42.4)	31 (37.3)	44 (43.6)

*APACHE-II* Acute Physiology and Chronic Health Evaluation-II, *BMI* body mass index, *CLSI* Clinical and Laboratory Standards Institute, *CPIS* Clinical Pulmonary Infection Score, *CRP* C-reactive protein, *MDR* multidrug-resistant, *mITT* modified intent-to-treat population, *PCR CT* polymerase chain reaction cycle threshold, *PDR* pan-drug-resistant, *SD* standard deviation, *SOFA* Sequential Organ Failure Assessment, *XDR* extensively drug resistant. ^†^Preventive ventilator-associated pneumonia measures (bundles) were elevation of the head of the bed, daily sedation vacations and extubation readiness assessment, peptic ulcer disease prophylaxis, deep vein thrombosis prophylaxis, and daily oral care with chlorhexidine

^*^n = 15; ^†^*n* = 82; ^‡^*n* = 82; ^§^*n* = 179; ^¶^*n* = 180 ^#^*n* = 81; **n = 177; ^††^*n* = 82; ^‡‡^minimum inhibitory concentrations were determined by CLSI broth microdilution at a centralised laboratory; ^§§^MDR, PDR, and XDR as defined by;[29] ^¶¶^subjects with PA-positive culture results but missing minimum inhibitory concentration records; ^##^all randomised subjects were positive for PA by PCR within 36 h before randomisation, but not all had positive cultures; ***n = 83; ^†††^*n* = 81; ^‡‡‡^*n* = 99; ^§§§^*n* = 12; ^¶¶¶^*n* = 77; ^###^*n* = 74; ****n = 89; ^††††^*n* = 76; ^‡‡‡‡^*n* = 92

### Randomisation and masking

Per protocol, subjects were randomised (1:1:1) to a single intravenous (IV) dose of MEDI3902 500 mg, 1500 mg, or placebo. Based on previous studies [19, 20] and pharmacokinetic (PK) data [21] received after the start of the study, a single MEDI3902 500 mg dose was not expected to maintain a target level of 1.7 μg/mL (derived from a murine model of PA pneumonia where mice were inoculated with PA 1 × LD100, data on file) for 21 days, and enrolment in this arm was discontinued after 16 subjects were dosed. Interim PK confirmed MEDI3902 1500 mg maintained the target level in 80% of patients through day 21 [21]. Subsequently, following protocol and statistical analysis plan (SAP) amendment, subjects were randomised (1:1) to MEDI3902 1500 mg or placebo (Additional file 1: Fig. S1), stratified by geographical region and duration of anti-PA antibiotic treatment within 96 h before randomisation (no antibiotic use, duration of no more than 72 h, duration > 72 h [except for systemic or aerosolised colistin; see exclusion criteria]). Subjects were followed until the end of the study period (day 50). An interactive web response system was used for randomisation to the treatment group and assignment of blinded investigational product kit numbers. To complete screening and ensure uniformity of inclusion criteria across sites, the eligibility of all potential patients was confirmed by the Clinical Coordinating Centre (Saint-Luc University Hospital, Brussels, Belgium). MEDI3902 and placebo were administered in a blinded fashion, and neither the subjects, their legal representatives, nor the investigators and sponsor staff involved in the treatment or clinical assessment of subjects were aware of the treatment received. The investigational products were handled by an unblinded investigational product manager at each site.

### Endpoints and assessments

The primary efficacy endpoint was the incidence of nosocomial PA pneumonia through 21 days post-dose in MEDI3902 1500 mg recipients versus placebo as determined by a blinded independent Endpoint Adjudication Committee (EAC). The EAC included three experts in intensive care medicine and two radiologists that used prespecified, stringent and non-subjective criteria agreed upon by both the US Food and Drug Administration and the European Medicines Agency. Subjects must have met radiological (new or worsening infiltrate consistent with pneumonia on chest X-rays), clinical, and microbiological criteria concurrently to be diagnosed with PA pneumonia (Additional file 1: Methods). In subjects with suspected or confirmed pneumonia, tracheobronchitis, or bacteraemia, blood and respiratory specimens were collected, and chest X-rays were performed as clinically indicated, until clinical resolution. Primary safety endpoints included the incidence of treatment-emergent adverse events (TEAEs), serious AEs (SAEs), and AEs of special interest assessed through 49 days post-dose.

Secondary endpoints were MEDI3902 serum/ETA PK parameters and serum anti-drug antibody responses through 49 days post-dose. Blood samples were collected immediately before MEDI3902 dosing on day 1, at the end of infusion, and 8- and 24-h later and on days 4, 8, 15, 22, 29, and 50 of follow-up.

The Additional file 1: Methods include exploratory endpoints and sample collection details.

### Statistical analysis

Following protocol/SAP amendment, planned enrolment was approximately 286 subjects randomised (1:1) to MEDI3902 1500 mg or placebo. Given the exploratory nature of the study, power was calculated based on Poisson regression with robust variance comparing MEDI3902 versus placebo groups (two-sided, *α* = 0.2), assuming a placebo group PA pneumonia incidence of 20%, a relative reduction of 50%, at least 80% power, and 20% adjustment for attrition. A relative reduction of 50% was considered clinically meaningful based on expert advice and published data [6].

Recruitment was stopped early after 168 patients were included (MEDI3902 1500 mg: *n* = 85; placebo: *n* = 83) due to slow enrolment.

Efficacy and PK were assessed in the modified intent-to-treat (mITT) population (all subjects randomised and dosed, analysed by randomised treatment). Following discontinued enrolment in the MEDI3902 500 mg arm and protocol/SAP amendment, efficacy was assessed in MEDI3902 1500 mg versus placebo recipients. The primary endpoint of nosocomial PA pneumonia was assessed by relative risk reduction (RRR), defined as 1—relative risk, and its corresponding two-sided 80% confidence interval (CI), as estimated from a Poisson regression model with robust variance and treatment group as a covariate (two-sided, *α* = 0.2). A positive RRR denoted less PA pneumonia in the MEDI3902 1500 mg group compared to placebo, and a negative RRR had more PA pneumonia in the MEDI3902 group. Patients with mixed culture results that included PA were counted towards the primary endpoint. Early discontinuation due to death from underlying disease was expected as the main cause of missing data. If no PA pneumonia occurred before discontinuation, the subject was considered as having no PA pneumonia infection in the primary efficacy analysis. No other imputation was applied to this analysis. Time to diagnosis of PA pneumonia, as judged by the EAC, was estimated by use of the Kaplan–Meier method. Safety was assessed in the as-treated population (all subjects randomised and dosed, analysed by treatment received). Safety and PK analyses included the 16 subjects who received MEDI3902 500 mg. Data were summarised descriptively, with no multiplicity adjustments.

### Post hoc analyses

An adjusted post hoc analysis of the primary efficacy endpoint was done to address possible selection bias due to an imbalance in baseline covariates including ECMO, PA-positive cultures, absolute neutrophil counts (ANC) and procalcitonin (PCT) levels. The impact of key baseline covariates on the RRR of PA pneumonia and all-cause mortality in MEDI3902 1500 mg recipients versus placebo, and on MEDI3902 serum PK, was also assessed (Additional file 1: Methods). Efficacy data were calculated using PCT and ANC quartiles. The groups with high and statistically significant RRR with baseline levels of ≤ 0.55 μg/L for procalcitonin and ≤ 8.17 × 10^3^ cells/μL for ANC correspond to combined quartiles 1–3 for procalcitonin and combined quartiles 1–2 for ANC.

### Role of the funding source

The study sponsor was involved in study design, data collection, data analysis, and data interpretation, with input from the authors, and in the writing of the report.

## Results

### Subjects

**Fig. 1:**
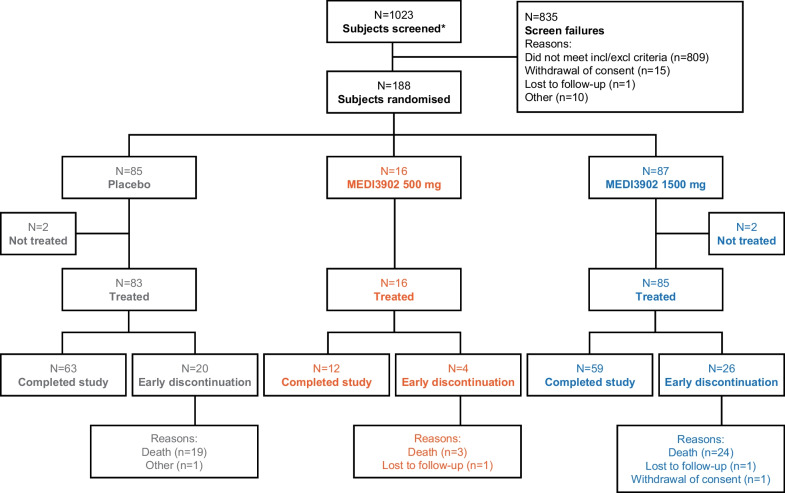
Subject disposition. *Subjects signed informed consent

Subjects were randomised between 13 April 2016 and 17 October 2019; the study was completed on 4 December 2019. Of the 1023 subjects screened, 835 (81.6%) could not be included in the study (Fig. 1). Overall, 184 randomised subjects received MEDI3902 1500 mg (*n* = 85), 500 mg (*n* = 16), or placebo (*n* = 83) (Fig. 1); 134/184 (72.8%) subjects completed the study, including 59 (69.4%) MEDI3902 1500 mg, 12 (75.0%) 500 mg, and 63 (75.9%) placebo recipients. The most frequent reason for discontinuation was death in three (18.8%), 24 (28.2%), and 19 (22.9%) subjects in the 500 mg, 1500 mg and placebo groups, respectively.

Baseline demographics, clinical severity scores, and Clinical and Laboratory Standards Institute (CLSI) PA antibiotic susceptibility results were generally similar between groups (Table 1). However, there was a numerically higher percentage of non-MDR PA-positive cultures in the MEDI3902 1500 (44.6%) mg group versus placebo (30.5%). Subjects in the MEDI3902 arms also had higher ANC (13.2 vs. 11.4 × 103 cells per microliter and PCT levels (1.0 vs. 0.61 μg/L) at baseline versus placebo (Table 1). All five components of the VAP prevention bundles were implemented in 42.4% and 37.3% of MEDI3902 and placebo recipients, respectively (Table 1 and Additional file 1: Table S3).

### Efficacy

The PA pneumonia incidence was 19/85 (22.4%) in MEDI3902 1500 mg recipients and 15/83 (18.1%) in placebo recipients; RRR − 23.7% (80% CI − 83.8%, 16.8%; *p* = 0.49) (Table 2). The primary endpoint was not met. Similar results were observed regarding exploratory efficacy endpoints, including all-cause pneumonia, all-cause pneumonia or death, antibiotic usage and duration of mechanical ventilation (Table 2 and Additional file 1: Results and Table S4). Specifically, when patients who died before study completion without PA pneumonia were classified as failure (and not as success as done in the primary analysis), the incidence of all-cause pneumonia or death was 30/85 (35.3%) in MEDI3902 1500 mg recipients and 22/83 (26.5%) in placebo recipients; RRR − 33.3% (80% CI − 79.8%, 1.4%). The results of the time-to-event analysis are shown in the Additional file (Additional file 1: Fig. S2), with most PA pneumonia occurring 15 days after randomisation in both groups. No significant difference in the incidence of other serious PA infections (tracheobronchitis, bacteraemia, intraabdominal or deep skin and soft tissue infections) was observed between the placebo group and the MEDI3902 1500 mg group (Additional file 1: Table S5). To make sure that decisions made by the EAC were in agreement with those made by the physicians at the bedside, the number of subjects with an adverse event of PA pneumonia, not confirmed as PA pneumonia by the EAC, but who received anti-PA antibiotics, was tabulated by the treatment group (Additional file 1: Table S6). Clinicians detected and treated with antibiotics only three and 0 additional events of potential PA pneumonia in placebo recipients and MEDI3902 1500 mg recipients, respectively.

**Table 2:** MEDI3902 phase II EVADE: efficacy through study day 22 (mITT)

	MEDI3902 500 mg (*N* = 16)	MEDI3902 1500 mg (*N* = 85)	Placebo (*N* = 83)	RRR^a^	80% CI^a^	*P* value^a^
Primary endpoint						
*P. aeruginosa* Pneumonia	2 (12.5%)	19 (22.4%)	15 (18.1%)	− 23.7%	− 83.8 to 16.8%	0.491
Exploratory endpoints of interest (FDA)						
All-cause pneumonia^b^	3 (18.8%)	25 (29.4%)	17 (20.5%)	− 43.6%	− 104 to -1.1%	0.186
All-cause pneumonia or death^c^	4 (25.0%)	30 (35.3%)	22 (26.5%)	− 33.2%	− 79.8 to 1.4%	0.222

RRR: relative risk reduction (a negative RRR denotes more PA pneumonia in the MEDI3902 1500 mg group compared to placebo); mITT = modified intent-to-treat population

All pneumonias were determined by an adjudication committee

^a^Relative risk reduction (MEDI3902 1500 mg vs. placebo), 80% confidence interval (CI), and *p* value based on Poisson regression with robust variance

^b^All-cause pneumonia: *P. aeruginosa* pneumonia or non-*P. aeruginosa* pneumonia occurring through study day 22

^c^All-cause pneumonia or death: *P. aeruginosa* pneumonia, non-*P. aeruginosa* pneumonia, or death occurring through study day 22

Additional post hoc analyses were done on baseline covariates (Additional file 1: Fig. S3). PCT and ANC had the greatest effect on RRR in PA pneumonia. At baseline, 115 patients (68% of the study population) had PCT levels below 0.55 μg/L. In that group of patients with a low PCT level, the incidence of PA pneumonia was 23.7% in the placebo and 12.5% in MEDI3902 1500 mg groups, corresponding to an 47.3% RRR (80% CI: 6.1% to 69.9%; *p* = 0.135). Similar results were observed in the subgroup of 83 patients (49% of the study population) with an ANC less than 8.17 × 103 cells/µL: PA pneumonia incidence was 17% and 2.8% in the placebo and MEDI3902 1500 mg group, respectively, corresponding to a 83.6% RRR (80% CI 39.5%, 95.5%; *P* = 0.038) (Additional file 1: Tables S7A and S7B). In subjects with ANC ≤ 8.17 × 103/µL, MEDI3902 treatment was associated with 56.5% RRR in all-cause mortality (80% CI 11.9%, 79.4%; *P* = 0.110). Conversely, RRRs were negative in patients with PCT levels above 0.55 μg/L and those with ANC above 8.17 × 103 cells/µL, denoting more PA pneumonia in the MEDI3902 group than in the placebo group (Additional file 1: Tables S7A and S7B).

Healthcare resource utilisation through 21 days post-dose for MEDI3902 1500 mg and placebo subjects with PA pneumonia were similar, including duration of mechanical ventilation (17.5 vs. 19.7 days, respectively), systemic antibiotic use (12.3 vs. 14.4 days) and duration of ICU stay (20.6 vs. 22.0 days) (Additional file 1: Table S4). Severity scores on day 1 of pneumonia onset and day of resolution, as well as changes in cellular and protein markers of inflammation, also did not demonstrate any significant differences between MEDI3902 patients and placebo patients with PA pneumonia (Additional file 1: Tables S8 and S10). The results of other exploratory analyses are described in the Additional file 1: Results, Tables S4 and S8-11 and Fig. S4.

### Pharmacokinetics

**Fig. 2:**
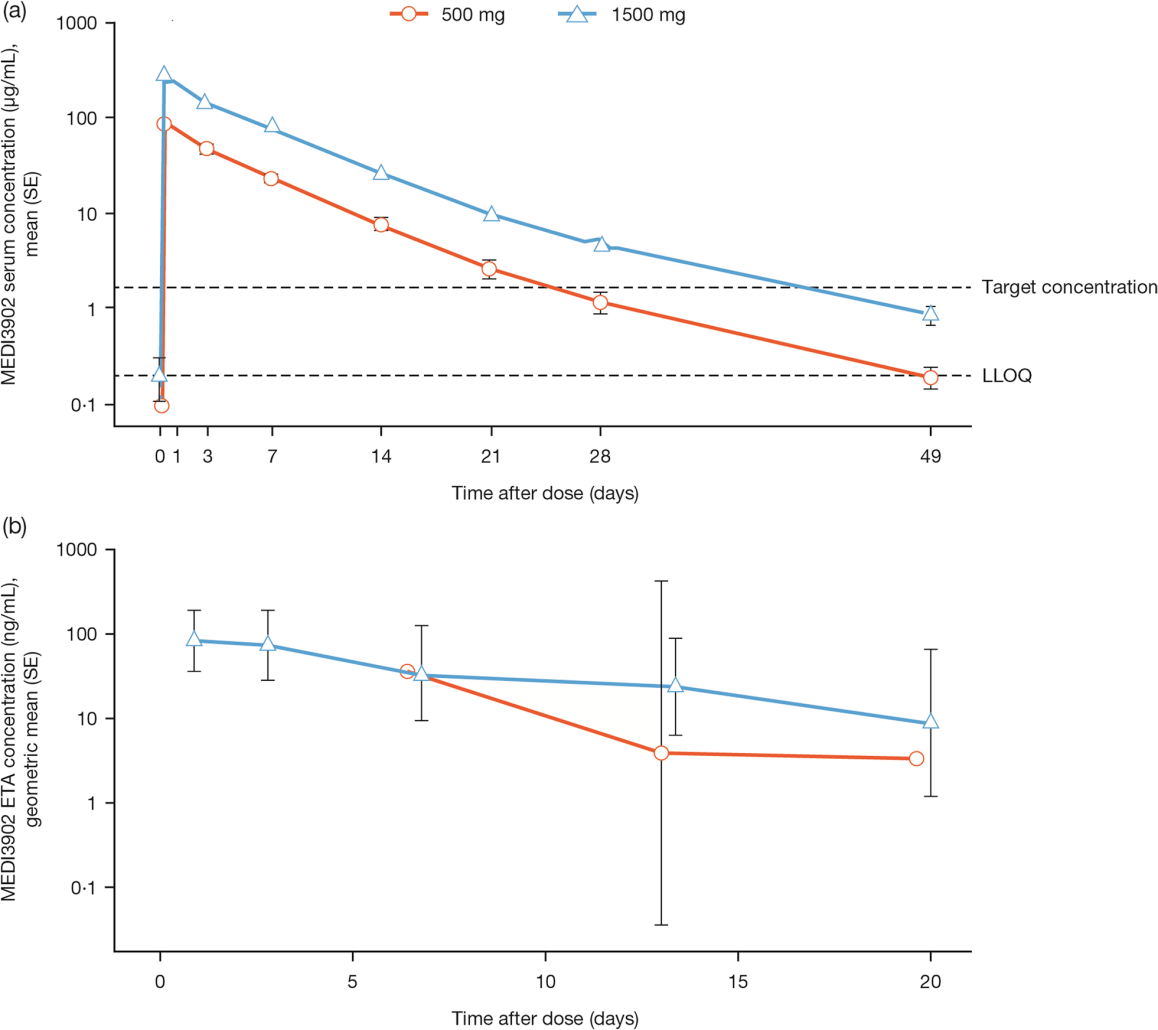
Concentration–time profile following a single IV dose of MEDI3902 500 mg and 1500 mg in serum (**a**) and endotracheal aspirate (**b**) PK geometric mean profile. ETA = endotracheal aspirate. IV = intravenous. LLOQ = lower limit of quantification. PK = pharmacokinetic. SE = standard error

**Fig. 3:**
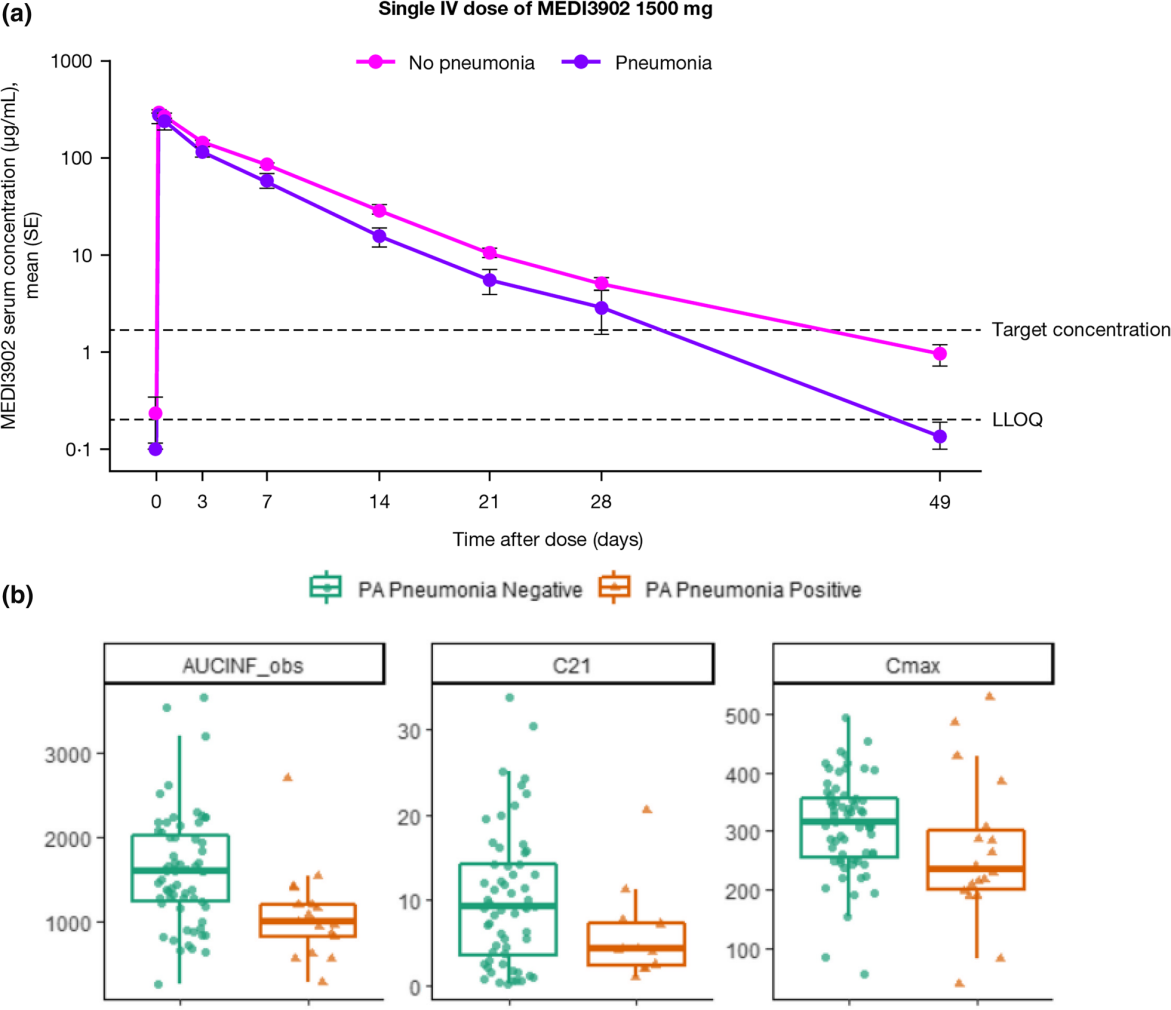
a Mean (SE) serum MEDI3902 concentrations in subjects with or without PA pneumonia. LLOQ = lower limit of quantification. SE = standard error. **b** MEDI3902 area under the curve from 0 to 21 days and concentrations obtained after a single IV dose of 1500 mg in patients with and without PA pneumonia. Cmax = maximal observed concentration; C_21_ = concentration 21 days post-dosing; AUC_0to21_ = Area under the curve from 0 to 21 days post-dose

Mean serum concentration–time profiles of MEDI3902 are shown in Figs. 2a and 3a, b. At 21 days following a 1500 mg infusion, the mean (standard deviation [SD]) serum MEDI3902 concentration was 9.46 (7.91) μg/mL, with 80.6% (*n* = 58/72) subjects achieving concentrations > 1.7 μg/mL, the target level (Table 3). In ETA, the geometric mean (SD) MEDI3902 concentration was 8.73 (± 11) ng/mL at day 21 (Fig. 2b and Additional file 1: Fig. S5); however, significant correlations were not observed between MEDI3902 concentrations in serum and ETA (*R*^2^ = 0.000081; data not shown). MEDI3902 PK parameters in serum and ETA are summarised in Table 3. Mean clearance from serum was 1.27 L/day, with a mean half-life of 5.65 days following a 1500 mg infusion. On day 2, the median MEDI3902 ETA/serum PK ratio was 0.06%, increasing to 0.1% on day 4. A positive exposure–response relationship was observed after a 1500 mg IV infusion, with numerically higher serum MEDI3902 concentrations observed in subjects who did not develop PA pneumonia versus those who did (Fig. 3a, b and Additional file 1: Fig. S6).

**Table 3:** MEDI3902 PK parameters

PK parameter	MEDI3902 500 mg (*N* = 16)		MEDI3902 1500 mg (*N* = 85)	
	*n*	Mean (SD)	*n*	Mean (SD)
*Serum*				
*C*_max,_ µg/mL	16	87.6 (23.9)	84	299 (94.1)
*C*_21_, µg/mL	14	2.56 (2.23)	72	9.46 (7.91)
AUC_0–21,_ day*µg/mL	16	418 (126)	81	1410 (599)
AUC_∞,_ day*µg/mL	16	440 (135)	81	1510 (675)
CL, L/day	16	1.31 (0.64)	81	1.27 (0.86)
*t*_½_, day	16	6.56 (4.03)	81	5.65 (2.69)

	*n*/N	%	*n*/N	%
Subjects with serum MEDI3902 levels > target level of 1.7 µg/mL on day 21	7/14	50.0%	58/72	80.6%
*Endotracheal aspirate*^†^				
*C*_max,_ µg/mL	–	–	n/a	0.083
AUC_0–21,_ day*µg/mL	–	–	n/a	0.704
AUC_∞,_ day*µg/mL	–	–	n/a	0.784
*t*_½_, day	–	–	n/a	6.3

AUC_0–21_ = the area under the concentration–time curve from time zero to 21 days post-dose. AUC_**∞**_ = the area under the concentration–time curve from zero to infinity. CL = clearance. *C*_max_ = maximum observed concentration. *C*_21_ = concentration 21 days post-dose. IV = intravenous. n/a = not applicable. SD = standard deviation.* t*_½_ = half-life

^†^Due to the large variability in measured endotracheal aspirate concentrations and limited samples per subject, it was not plausible to calculate endotracheal aspirate PK parameter at an individual level. Therefore, endotracheal aspirate PK parameters were calculated based on geometric mean concentration–time profile

### Serum anti-drug antibodies

Serum anti-drug antibodies were detected at baseline in 2/85 (2.4%) subjects in the MEDI3902 1500 mg group, 2/16 (12.5%) in the 500 mg group, and 3/83 (3.6%) in the placebo group. The number of subjects with persistent serum anti-drug antibodies (defined as a positive test of at least 2 or the last post-baseline assessment) was similar between the MEDI3902 1500 mg (*n* = 4/85; 4.9%) and placebo (*n* = 4/83; 5.1%) groups. No differences were observed in MEDI3902 PK or safety profiles in subjects with and without post-baseline serum anti-drug antibodies responses.

### Safety

The incidence of TEAEs, SAEs, and TEAEs of > grade 3 severity reported through 49 days post-dose was similar between MEDI3902 1500 mg and placebo groups (Table 4). TEAEs related to treatment were reported in three subjects receiving MEDI3902 1500 mg (sinus tachycardia [*n* = 1, grade 1, resolved the same day] and infusion-related reactions [*n* = 2, grade 1 and 3, resolved during the study]) and in one placebo recipient (acute kidney injury, grade 4). No treatment-related SAEs were reported.

**Table 4:** Overall summary of TEAEs through 49 days post-dose

Subjects,* *n* (%) with ≥ 1 event	MEDI3902 500 mg (*N* = 16)	MEDI3902 1500 mg (*N* = 85)	Placebo (*N* = 83)
TEAE	15 (93.8)	84 (98.8)	81 (97.6)
Treatment-related TEAE	0 (0.0)	3 (3.5)	1 (1.2)
TEAE of ≥ grade 3 severity^†^	12 (75.0)	60 (70.6)	54 (65.1)
Death (grade 5 severity†)	3 (18.8)	24 (28.2)	19 (22.9)
Serious^‡^ TEAE	4 (25.0)	38 (44.7)	35 (42.2)
Serious^‡^ and/or ≥ grade 3 severity^†^ TEAE	12 (75.0)	60 (70.6)	55 (66.3)
Treatment-related serious^‡^ TEAE	0 (0.0)	0 (0.0)	1 (1.2)
TEAE leading to discontinuation of treatment	0 (0.0)	0 (0.0)	0 (0.0)
AESI^§^	0 (0.0)	2 (2.4)	1 (1.2)
Treatment-related AESI^§^	0 (0.0)	2 (2.4)	0 (0.0)
AESI^§^ of ≥ grade 3 severity^†^	0 (0.0)	1 (1.2)	1 (1.2)

*AE *adverse event, *AESI* adverse event of special interest, *TEAE* treatment-emergent adverse event

^*^Subjects are counted once for each category regardless of the number of events; ^†^grade 3, severe; grade 4, life-threatening; grade 5, fatal; ^‡^serious adverse event criteria: death, life-threatening, required inpatient hospitalisation, prolongation of existing hospitalisation, persistent or significant disability/incapacity, important medical event, congenital anomaly/disorder (in the offspring of the subject); ^§^AESI defined as targeted AEs of hepatic function abnormalities, hypersensitivity reactions (including anaphylaxis), infusion-related reactions, and immune complex disease (including vasculitis, endocarditis, neuritis, and glomerulonephritis)

Overall, 46 deaths were reported until 49 days post-dose. A slightly higher number of deaths in the MEDI3902 1500 mg group (*n* = 24/85; 28.2%) versus placebo (*n* = 19/83; 22.9%; Table 4) was not statistically significant (RRR [80% CI] − 23.3% [− 73.3, 12.2%]; *p* = 0.43). No deaths were considered related to MEDI3902, as adjudicated by the blinded investigators and independent unblinded DSMB.

## Discussion

The primary efficacy endpoint of reduction in PA pneumonia incidence, with a relative reduction of 50% considered clinically meaningful based on expert advice and published data [6], was not achieved. However, the certainty of our overall study findings may be limited given that the study was interrupted prematurely before the target number of patients was reached due to slow enrolment, and thus, the analyses may be underpowered. A single IV dose of MEDI3902 1500 mg provided PK serum exposure above the target level of 1.7 µg/mL for at least 21 days in most subjects, with MEDI3902 concentrations detectable in the ETA. The incidence of persistent serum anti-drug antibodies was low, occurring in < 5% of MEDI3902 1500 mg recipients. A positive exposure–response relationship was observed for the MEDI3902 1500 mg group, with a greater MEDI3902 area under the concentration–time curve from time zero to 21 days post-dose associated with a lower probability of PA pneumonia.

For mechanically ventilated critically ill subjects, high baseline inflammatory status or cachexia increases protein catabolism and volume distribution of many drugs [22], potentially lowering MEDI3902 exposure and increasing PA pneumonia susceptibility simultaneously. A higher MEDI3902 dose and/or direct administration into the tracheobronchial tree by aerosolisation may be considered for the most seriously ill subjects to achieve protection, especially since MEDI3902 ETA/serum PK ratio was low (0.1% on day 4).

An imbalance in baseline inflammation was reflected in PCT and ANC levels. Subjects with lower baseline PCT or ANC had slightly higher mean PK levels and may exhibit a greater MEDI3902 treatment response versus placebo against PA pneumonia. These results provide important lessons on pathogen-specific PA pneumonia complexity in ICU trials with potential implications for future study design. Whereas APACHE-II scores have been used as eligibility criteria to avoid enrolling the sickest patients [23], clinicians might also consider baseline levels of certain biomarkers as exclusion criteria and/or for stratifying patients by severity at randomisation, particularly in pre-emptive treatment studies of colonised patients. While PCT and ANC levels may not correlate directly with PA pneumonia, these markers may identify patients with higher bacterial load and higher inflammatory status, and therefore a higher risk of pneumonia. [24–26]. Conversely, patients with higher levels of these biomarkers (regardless of apparent pathogen levels) may be too sick to benefit from treatment, or may have progressed too far in the development of symptomatic pneumonia [27]. Whether MEDI3902 could have increased the rate of PA pneumonia in patients with high inflammatory status in the present study remains highly speculative. However, some potential deleterious effects of monoclonal antibodies have already been reported in other studies, including a large randomised trial having assessed the benefit of a combination of casirivimab and imdevimab given together in patients admitted to the hospital with COVID-19. Although the monoclonal combination improved survival and other clinical outcomes in patients who did not have detectable anti-SARS-CoV-2 antibodies (i.e. had not yet mounted their own humoral immune response), no clinical benefit was observed in patients who were seropositive at baseline, suggesting the possibility of a conflict between the subjects' own immune defences and the monoclonal antibodies [28]. Further research is required to understand the role of these surrogate biomarkers for the inflammatory response in risk stratification and treatment decisions.

The safety profile of MEDI3902 was acceptable, with a similar incidence of TEAEs and SAEs in the MEDI3902 1500 mg and placebo groups. TEAEs were generally reflective of the critically ill patient population and the numerical imbalance in deaths across treatment groups was expected due to the observed imbalance in baseline disease characteristics.

## Limitations

This study has several limitations. First, the trial did not achieve its planned sample size, with the recruitment being stopped early due to low enrolment and thus was underpowered to detect small but clinically important treatment effects in the entire study population, as well as in specific subgroups of patients. The main contributing factor of low recruitment was that the proportion of patients meeting all the eligibility criteria was lower than expected, which resulted in an average of 4 patients randomised per site. Other major contributing factors included the unusually complex screening process and the difficulty for the attending clinicians to distinguish patients who were actually infected with PA from those only colonised, often resulting in the immediate administration of new antibiotics and making patients ineligible for randomisation. Second, the investigators may not have screened some eligible patients either because of the absence of consent or difficulties in identifying patients potentially colonised by PA. Since this number was not recorded, the extent of this bias cannot be ascertained. Third, the patient population was primarily Western European, thus reflecting the MDR profile from one region and the absence of strict guidelines for antibiotic treatment resulted in heterogeneity in antibiotic use. Fourth, colonisation, as determined by PCR, may not coincide with a positive PA culture and, since colonisation assays were not quantitative, active infection (rather than just colonisation) could not be ruled out at the time of treatment. Fifth, misclassification of pneumonia is also possible due to difficulty in diagnosis, particularly once new antibiotics were prescribed. However, the trial was double-blinded and a strict protocol was applied to diagnose PA pneumonia. Sixth, MEDI3902 endotracheal aspirate concentrations were rather low questioning whether adequate epithelial lining fluid levels of the monoclonal antibody were present in these patients to prevent infection*.* Efficacy may have increased with higher antibody doses, which were not assessed in this study. Furthermore, results derived from post hoc analyses should be interpreted with caution.

## Conclusions

Among ICU patients requiring prolonged mechanical ventilation and colonised by PA as detected by PCR, a single intravenous dose of 1500 mg of the bivalent, bispecific monoclonal antibody MEDI3902 (gremubamab) was safe, with a number of AEs similar to placebo, but it did not reduce PA nosocomial pneumonia incidence. Whether MEDI3902 may help prevent PA pneumonia, with higher doses or in more specific patient populations, would require additional studies.

## Supplementary Information

**Additional file 1: **Supplementary Methods, Results, Tables and Figures.

## Data Availability

Data underlying the findings described in this manuscript can be obtained in accordance with AstraZeneca’s data sharing policy.

## Abbreviations

AE: Adverse event
ANC: Absolute neutrophil count
APACHE-II: Acute Physiology and Chronic Health Evaluation-II
BAL: Bronchoalveolar lavage
CI: Confidence interval
CPIS: Clinical pulmonary infection score
EAC: Endpoint Adjudication Committee
ETA: Endotracheal aspirate
ICU: Intensive care unit
IV: Intravenous
MDR: Multidrug-resistant
mITT: Modified intent-to-treat
PA: Pseudomonas aeruginosa
PCR: Polymerase chain reaction
PCT: Procalcitonin
PK: Pharmacokinetic
RRR: Relative risk reduction
SAE: Serious adverse event
SAP: Statistical analysis plan
SOFA: Sequential Organ Failure Assessment
TEAE: Treatment-emergent adverse event
VAP: Ventilator-associated pneumonia

## References

[1] Ramírez-Estrada S, Borgatta B, Rello J (2016). *Pseudomonas aeruginosa* ventilator-associated pneumonia management. Infect Drug Resist.

[2] Kollef MH, Chastre J, Fagon JY, Francois B, Niederman MS, Rello J (2014). Global prospective epidemiologic and surveillance study of ventilator-associated pneumonia due to *Pseudomonas aeruginosa*. Crit Care Med.

[3] Jones RN (2010). Microbial etiologies of hospital-acquired bacterial pneumonia and ventilator-associated bacterial pneumonia. Clin Infect Dis.

[4] Micek ST, Wunderink RG, Kollef MH, Chen C, Rello J, Chastre J (2015). An international multicenter retrospective study of *Pseudomonas aeruginosa* nosocomial pneumonia: impact of multidrug resistance. Crit Care.

[5] Tumbarello M, De Pascale G, Trecarichi EM, Spanu T, Antonicelli F, Maviglia R (2013). Clinical outcomes of *Pseudomonas aeruginosa* pneumonia in intensive care unit patients. Intensive Care Med.

[6] François B, Luyt CE, Dugard A, Wolff M, Diehl JL, Jaber S (2012). Safety and pharmacokinetics of an anti-PcrV PEGylated monoclonal antibody fragment in mechanically ventilated patients colonized with *Pseudomonas aeruginosa*: a randomized, double-blind, placebo-controlled trial. Crit Care Med.

[7] François B, Chastre J, Eggiman P, Laterre PF, Torres A, Sanchez M (2016). The SAATELLITE and EVADE clinical studies within the COMBACTE consortium: a public-private collaborative effort in designing and performing clinical trials for novel antibacterial drugs to prevent nosocomial pneumonia. Clin Infect Dis.

[8] Torres A, Niederman MS, Chastre J, Ewig S, Fernandez-Vandellos P, Hanberger H (2017). International ERS/ESICM/ESCMID/ALAT guidelines for the management of hospital-acquired pneumonia and ventilator-associated pneumonia: guidelines for the management of hospital-acquired pneumonia (HAP)/ventilator-associated pneumonia (VAP) of the European Respiratory Society (ERS), European Society of Intensive Care Medicine (ESICM), European Society of Clinical Microbiology and Infectious Diseases (ESCMID) and Asociación Latinoamericana del Tórax (ALAT). Eur Respir J.

[9] Ferrer R, Martin-Loeches I, Phillips G, Osborn TM, Townsend S, Dellinger RP (2014). Empiric antibiotic treatment reduces mortality in severe sepsis and septic shock from the first hour: results from a guideline-based performance improvement program. Crit Care Med.

[10] American Thoracic Society, Infectious Diseases Society of America. Guidelines for the management of adults with hospital-acquired, ventilator-associated, and healthcare-associated pneumonia. Am J Respir Crit Care Med. 2005;171(4):388–416. 10.1164/rccm.200405-644ST 15699079

[11] Pang Z, Raudonis R, Glick BR, Lin TJ, Cheng Z (2019). Antibiotic resistance in *Pseudomonas aeruginosa*: mechanisms and alternative therapeutic strategies. Biotechnol Adv.

[12] Kalil AC, Metersky ML, Klompas M, Muscedere J, Sweeney DA, Palmer LB (2016). Management of adults with hospital-acquired and ventilator-associated pneumonia: 2016 clinical practice guidelines by the Infectious Diseases Society of America and the American Thoracic Society. Clin Infect Dis.

[13] Tümmler B. Emerging therapies against infections with *Pseudomonas aeruginosa*. F1000Res. 2019;8(F1000 Faculty Rev-1371):1371.

[14] Que YA, Lazar H, Wolff M, François B, Laterre PF, Mercier E (2014). Assessment of panobacumab as adjunctive immunotherapy for the treatment of nosocomial *Pseudomonas aeruginosa* pneumonia. Eur J Clin Microbiol Infect Dis.

[15] DiGiandomenico A, Keller AE, Gao C, Rainey GJ, Warrener P, Camara MM (2014). A multifunctional bispecific antibody protects against *Pseudomonas aeruginosa*. Sci Transl Med..

[16] Ali SO, Yu XQ, Robbie GJ, Wu Y, Shoemaker K, Yu L, et al. Phase 1 study of MEDI3902, an investigational anti-*Pseudomonas aeruginosa* PcrV and Psl bispecific human monoclonal antibody, in healthy adults. Clin Microbiol Infect. 2019;25(5):629.e1–.e6. 10.1016/j.cmi.2018.08.004 30107283

[17] Le HN, Tran VG, Vu TTT, Gras E, Le VTM, Pinheiro MG (2019). Treatment efficacy of MEDI3902 in *Pseudomonas aeruginosa* bloodstream infection and acute pneumonia rabbit models. Antimicrob Agents Chemother.

[18] Le HN, Quetz JS, Tran VG, Le VTM, Aguiar-Alves F, Pinheiro MG (2018). MEDI3902 correlates of protection against severe *Pseudomonas aeruginosa* pneumonia in a rabbit acute pneumonia model. Antimicrob Agents Chemother.

[19] Francois B, Jafri HS, Chastre J, Sanchez-Garcia M, Eggimann P, Dequin PF (2021). Efficacy and safety of suvratoxumab for prevention of *Staphylococcus aureus* ventilator-associated pneumonia (SAATELLITE): a multicentre, randomised, double-blind, placebo-controlled, parallel-group, phase 2 pilot trial. Lancet Infect Dis.

[20] Yu XQ, Robbie GJ, Wu Y, Esser MT, Jensen K, Schwartz HI (2017). Safety, tolerability, and pharmacokinetics of MEDI4893, an investigational, extended-half-life, anti-staphylococcus aureus alpha-toxin human monoclonal antibody. Healthy Adults Antimicrob Agents Chemother.

[21] Guo X, François B, Bourgeois M, Komnos A, Rozgonyi ZD, Ali SA, et al. Interim pharmacokinetic analysis from the EVADE phase-2 clinical trial of MEDI3902, a bispecific monoclonal antibody against PcrV and Psl of *Pseudomonas aeruginosa*. In: 2018 E, editor. 28th European congress of clinical microbiology and infectious disease; 21–24 April 2018; Madrid, Spain: ECCMID; 2018. p. P2213.

[22] O'Leary-Kelley C, Bawel-Brinkley K (2017). Nutrition support protocols: enhancing delivery of enteral nutrition. Crit Care Nurse.

[23] Zeng J, Wang CT, Zhang FS, Qi F, Wang SF, Ma S (2016). Effect of probiotics on the incidence of ventilator-associated pneumonia in critically ill patients: a randomized controlled multicenter trial. Int Care Med.

[24] Marik PE, Stephenson E (2020). The ability of procalcitonin, lactate, white blood cell count and neutrophil-lymphocyte count ratio to predict blood stream infection. Analysis of a large database. J Crit Care.

[25] Gregoriano C, Heilmann E, Molitor A, Schuetz P (2020). Role of procalcitonin use in the management of sepsis. J Thorac Dis.

[26] Huang Z, Fu Z, Huang W, Huang K (2020). Prognostic value of neutrophil-to-lymphocyte ratio in sepsis: a meta-analysis. Am J Emerg Med.

[27] Melsen WG, Rovers MM, Groenwold RHH, Bergmans DC, Camus C, Bauer TT (2013). Attributable mortality of ventilator-associated pneumonia: a meta-analysis of individual patient data from randomised prevention studies. Lancet Infect Dis.

[28] Group RC (2022). Casirivimab and imdevimab in patients admitted to hospital with COVID-19 (RECOVERY): a randomised, controlled, open-label, platform trial. Lancet (Lond Engl).

[29] Magiorakos AP, Srinivasan A, Carey RB, Carmeli Y, Falagas ME, Giske CG (2012). Multidrug-resistant, extensively drug-resistant and pandrug-resistant bacteria: an international expert proposal for interim standard definitions for acquired resistance. Clin Microbiol Infect.

