# Supplementary material for: Safety, efficacy, and pharmacokinetics of gremubamab (MEDI3902), an anti-Pseudomonas aeruginosa bispecific human monoclonal antibody, in P. aeruginosa-colonised, mechanically ventilated intensive care unit patients: a randomised controlled trial
Source: Crit Care. 2022 Nov 15;26:355. doi: 10.1186/s13054-022-04204-9 (PMC9666938; doi:10.1186/s13054-022-04204-9)
Supplement: Supplementary file 1 — Additional file 1: Supplementary Methods, Results, Tables and Figures. [file 13054_2022_4204_MOESM1_ESM.pdf]

Additional file

Safety, efficacy, and pharmacokinetics of gremubamab (MEDI3902), an anti *Pseudomonas aeruginosa* bispecific human monoclonal antibody, in *P. aeruginosa* colonised, mechanically ventilated intensive care unit patients: a randomised controlled trial

Jean Chastre, Bruno François, Marc Bourgeois, Apostolos Komnos, Ricard Ferrer, Galia Rahav, Nicolas De Schryver, Alain Lepape, Iftihar Koksal, Charles-Edouard Luyt, Miguel Sánchez-García, Antoni Torres, Philippe Eggimann, Despoina Koulenti, Thomas L. Holland, Omar Ali, Kathryn Shoemaker, Pin Ren, Julien Sauser, Alexey Ruzin, David E. Tabor, Ahmad Akhgar, Yuling Wu, Yu Jiang, Antonio DiGiandomenico, Susan Colbert, Drieke Vandamme, Frank Coenjaerts, Surbhi Malhotra-Kumar, Leen Timbermont, Antonio Oliver, Olivier Barraud, Terramika Bellamy, Marc Bonten, Herman Goossens, Colin Reisner, Mark T. Esser, and Hasan S. Jafri, on behalf of The COMBACTE-MAGNET EVADE Study Group

Corresponding author:

Professor Jean Chastre, Service de Médecine Intensive Réanimation, Institut de Cardiologie, Groupe Hospitalier Pitié-Salpêtrière, 47–83 Bd de l'Hôpital, 75651 Paris, France. Telephone: +33 615446324; Email: jean.chastre@aphp.fr

Alternate corresponding author:

Hasan S. Jafri, MD, Senior Director, Clinical Research and Development, Vaccines and Immune Therapies, AstraZeneca Biopharmaceuticals, One MedImmune Way, Gaithersburg, MD 20878, USA; Email: hasan.jafri.md@gmail.com

Journal: Intensive Care Medicine

Contents:

- **Supplementary Methods**
    - *Inclusion/exclusion criteria*
    - *Criteria for diagnosis of* P. aeruginosa *pneumonia*
    - *Post-hoc analyses*
    - *Exploratory endpoints*
    - *Specimen collection and schedule of study procedures for exploratory endpoints*
- **Supplementary Results**
    - *Exploratory efficacy analyses*
    - *Exploratory biomarker analyses*
- ***Supplementary table 1*: Number of randomised subjects by country**
- ***Supplementary table 2*: List of participating principal investigators by country and site**
- ***Supplementary table 3*: Ventilator-associated pneumonia prevention measures used in placebo and MEDI3902 patients (mITT)**
- ***Supplementary table 4*: Healthcare resource utilisation through 21 days post-dose for subjects with and without EAC-determined PA pneumonia**
- ***Supplementary table 5*: Incidence (n, %) of serious PA disease through 21 days post-dose by event attributable to PA**
- ***Supplementary table 6:* Subjects with PA pneumonia (not EAC-determined) with anti-PA effective antibiotics, which overlap the adverse event as-treated population**
- ***Supplementary tables 7A and 7B:* PA pneumonia incidence by procalcitonin and absolute neutrophil count quartiles at baseline – (mITT)**
- ***Supplementary table 8*: Severity scores (mean [standard deviation]) related to first EAC-determined PA pneumonia through 21 days post-dose in subjects with EAC-determined PA pneumonia**
- ***Supplementary table 9*: Change from baseline (mean [standard deviation]) in cellular and protein markers of inflammation through day 8 (all subjects, mITT)**
- ***Supplementary table 10*: Change from baseline in cellular and protein markers of inflammation through day of clinical resolution for subjects with EAC-determined *P. aeruginosa* pneumonia (mITT)**
- ***Supplementary table 11:* Subgroups by Baseline Disease (mITT)**
- ***Supplementary figure 1*: Study design.**
- ***Supplementary figure 2*: Cumulative incidence function for EAC-determined PA pneumonia with death as a completing risk (mITT).**
- ***Supplementary figure 3*: Impact of baseline covariates on MEDI3902 efficacy against PA pneumonia in the overall study (post-hoc analysis, mITT).**
- ***Supplementary figure 4*: Correlation between anti-cytotoxicity (a) or opsonophagocytic killing activity (b) and serum concentrations of MEDI3902 following 1500 mg dosing.**
- ***Supplementary figure 5*: MEDI3902 1500 mg endotracheal aspirate pharmacokinetic (PK) by visit.**
- ***Supplementary figure 6:* Exposure–response relationship between MEDI3902 1500 mg AUC_{0-21} and the likelihood of developing PA pneumonia.**

Supplementary Methods

Inclusion/exclusion criteria

Inclusion criteria

Subjects must have met all of the following criteria:

- Male or female 18 years of age or older at the time of study entry
- Written informed consent and any locally required authorisation obtained from the subject/legal representative before performing any protocol-related procedures, including screening assessments
- Females of childbearing potential who are sexually active with a non-sterilised male partner must have had evidence of not being pregnant upon enrolment and have a negative pregnancy test before the administration of the investigational product. Females of childbearing potential were defined as those who are not surgically sterile (i.e., bilateral oophorectomy or complete hysterectomy) or those who are not premenarchal or postmenopausal (defined as 12 months with no menses without an alternative medical cause)
- Currently intubated and on mechanical ventilation in the intensive care unit
- Tracheal sample collected and positive by polymerase chain reaction (PCR) for *Pseudomonas aeruginosa* (PA) within 36 hours before randomisation
- Expected to remain intubated and mechanically ventilated for at least 72 hours based on investigator estimate
- No diagnosis of new-onset pneumonia within 72 hours before randomisation (subjects with evidence of resolved pneumonia will be eligible)
- Expected to survive for >2 weeks based on investigator judgement
- Expected to participate in the study through 49 days post-dose

Exclusion criteria

Any of the following excluded the subject from participation in the study:

- Acute confirmed or suspected pseudomonal disease at study enrolment and investigational product dosing (endotracheal colonisation is acceptable and required [see inclusion criterion 5])
- Clinical Pulmonary Infection Score of at least 6 based on contributing parameters measured within the past 24 hours, before investigational product dosing
- Active pulmonary disease that would impair the ability to diagnose pneumonia, such as active tuberculosis or fungal disease, obstructing lung cancer, large pleural effusion or empyema, cystic fibrosis, or acute respiratory distress syndrome with lung “white out”
- Subjects who were tracheostomy-dependent before current hospital admission
- Receipt of anti-PA antibiotics (systemic colistin or aerosolised colistin) for > 72 hours within 96 hours before randomisation or anticipated ongoing receipt of the anti-PA antibiotics
- Burns >40% body surface area

- An Acute Physiology and Chronic Health Evaluation-II (APACHE-II) score of at least 25 or a Sequential Organ Failure Assessment (SOFA) score of at least 12 at the time of randomisation. Vasopressors only used to improve cerebral perfusion pressure (e.g., subarachnoid haemorrhage) were not to be used to calculate the cardiovascular component of the SOFA score

- Receipt of any investigational drug therapy within 30 days before investigational product dosing

- Previous receipt of a mAb

- Subjects with human immunodeficiency virus (HIV) infection who did not have well-controlled HIV infection in the investigator's opinion. Subjects with a history of HIV infection who had been on highly active antiretroviral therapy and were asymptomatic from HIV infection for at least 6 months were permitted to be enrolled

- Lymphoma not in complete remission and on chemotherapy

- Recipients of bone marrow, stem cell, or solid organ transplant who were not currently in complete remission

- Receipt of chemotherapy or other immunosuppressive drugs including glucocorticoid therapy (prednisone 20 mg or equivalent, daily or every other day for 30 days) in the previous 2 months

- History of known hypersensitivity to any component of the investigational product

- Pregnant or nursing female

Criteria for diagnosis of* P. aeruginosa *pneumonia

Subjects had to meet all the following three criteria (radiographic, clinical, and microbiological) to be diagnosed with *P. aeruginosa* pneumonia:

- Radiographic – new or worsening infiltrate consistent with pneumonia on chest X-ray, as diagnosed by a qualified radiologist within 24 hours of the event

- Clinical – at least two of the following minor or one major new-onset respiratory sign or symptom:

    - Minor criteria: at least one systemic sign of infection, including abnormal temperature (oral or tympanic temperature >38°C or a core temperature of <35°C or at least 38·3°C), and/or abnormal white blood cell (WBC) count (WBC count <4500 cells/mm^3, >10,000 cells/mm^3, or >15% band neutrophils); production of new purulent endotracheal secretions; new physical examination findings consistent with pneumonia/pulmonary consolidation such as auscultatory findings (e.g., rales, rhonchi, and bronchial breath sounds) or dullness to percussion

    - Additional minor criteria in subjects who were no longer mechanically ventilated included a new-onset or worsening cough, production of purulent sputum, pleuritic chest pain, dyspnoea, tachypnoea (respiratory rate >30 breaths/minute), or hypoxaemia defined as oxygen (O_2) saturation <90% or PaO2 <60 mm Hg on room air if lower than baseline, or a need to initiate or increase sustained (at least 3 hours) supplemental O_2 to maintain pre-event baseline saturations

    - Major criteria for mechanically ventilated subjects: acute changes made in the ventilatory support system to enhance oxygenation, as determined by a PaO_2/FiO_2 ratio <240 mm Hg

maintained for at least 4 hours or a decrease in PaO_2/FiO_2 by at least 50 mm Hg maintained for at least 4 hours

- Major criteria for subjects who were no longer mechanically ventilated: a need to initiate non-invasive or re-initiate invasive mechanical ventilation due to respiratory failure or worsening respiratory status

- Microbiologic confirmation by at least one of the following within 24 hours of the onset of the event:

  - Respiratory specimen positive for *P. aeruginosa* by culture of a respiratory secretion obtained by endotracheal aspiration or bronchoscopy in intubated subjects, by bronchoscopy in non-mechanically ventilated subjects (if obtained as part of necessary clinical management), and by expectorated sputum in subjects who were not intubated but met the protocol definition of mechanical ventilation

  - Blood culture positive for *P. aeruginosa* in the absence of a primary source of infection outside of the lung

  - Pleural fluid aspirate or lung tissue culture positive for *P. aeruginosa* during an episode of pneumonia if obtained as part of a subject's necessary clinical management

Post-hoc analyses

Adjusted post-hoc analysis of the primary efficacy endpoint

The adjusted analysis objective was to estimate the effect of MEDI3902 1500mg compared to placebo on the incidence of EAC-determined PA pneumonia within 21 days post-dose adjusted for baseline covariates. Inverse probability of treatment weighting was used to address possible selection bias due to baseline imbalance. The weights were computed for each patient using propensity score (PS). The PS represents the probability of a patient to be assigned to the intervention arm, given a set of explanatory variables. The analysis was adjusted for the following baseline variables:

- Age in years
- Gender (male or female)
- Body mass index in kg/m^2
- Pre-existing comorbidities (chronic obstructive pulmonary disease, congestive heart failure, coronary artery disease, angina, history of angioplasty, peripheral vascular disease, cerebrovascular disease, hypertension, chronic liver disease, diabetes, pulmonary artery hypertension, renal insufficiency, thoracic malignancy, and known immunodeficiencies)
- Reason for ICU admission (cardiovascular disorders, infection, neurologic disorders, post-operative, respiratory disease, and other)
- Duration of previous mechanical ventilation in days
- Previous episode of PA infections (yes or no)
- SOFA score
- PA bacterial load (Ct value)
- Procalcitonin concentration in µg/L
- C-reactive protein concentration in mg/dL
- Absolute neutrophil count in $10^3/\mu L$

A sensitivity analysis was done with the addition of the centre to the variables mentioned above. The PS was computed using generalised boosted regression. Weighted Poisson regression with robust variance was then applied to estimate the treatment effect. The weights are 1/PS for subjects in the intervention arm and 1/(1 – PS) for the subjects in the placebo arm, meaning that more weights are given to subjects that were less likely to be randomised to the arm they were. To account for missing data, multiple imputation was applied using 20 imputed datasets. The analyses were done using the

modified intention-to-treat (mITT) population, and results are shown for MEDI3902 1500 mg compared to placebo.

Post-hoc analyses of baseline covariates

The methodology and rationale for performing the post-hoc analyses followed the sequential process: i) we observed that the MEDI3902 arm appeared to have higher baseline inflammation compared with the placebo arm, with particularly higher WBC counts, absolute neutrophil counts, and procalcitonin concentrations in the MEDI3902 arm; ii) we then decided to examine the impact of the baseline covariates (such as previous mechanical ventilation duration, physiology scores, PCR Ct value as a measure of PA load, procalcitonin concentrations, absolute neutrophil counts, and WBC counts) on efficacy for PA pneumonia – we noticed that procalcitonin concentrations and absolute neutrophil counts had the greatest impact on the relative risk reduction (RRR) of PA pneumonia; iii) we then decided to perform the quartile analyses (a statistically sound way to examine the effect over the quantitative range) on the baseline procalcitonin concentrations and absolute neutrophil counts. The groups with high and statistically significant RRR with the baseline levels of $\leq 0{\cdot}55$ µg/L for procalcitonin and $\leq 8{\cdot}17\times10^3$ cells/µL for absolute neutrophil count correspond to combined quartiles 1–3 for procalcitonin and combined quartiles 1–2 for absolute neutrophil count.

Exploratory endpoints

Exploratory efficacy endpoints included: tracheal PA colonisation status through 21 days post-dose; incidence of all-cause nosocomial pneumonia through 21 days post-dose; incidence of PA tracheobronchitis through 21 days post-dose; incidence of nosocomial pneumonia caused by PA through 28 days post-dose; incidence of serious PA disease summarised through 21 days post-dose as measured by pneumonia, bacteraemia, intra-abdominal infection, deep skin or tissue infection, meningitis, or death attributable to PA; healthcare resource utilisation (days of hospital stay, days of ICU stay, days of mechanical ventilation, and number of days of antibiotic usage, number of chest computed tomography scans, and use of post-hospital discharge resources) through 21 and 49 days post-dose; severity of breakthrough nosocomial pneumonia caused by PA measured by days of mechanical ventilation, CPIS, and SOFA score through 21 days post-dose; all-cause mortality through 28 days post-onset of breakthrough nosocomial *P. aeruginosa* pneumonia; all-cause mortality through 49 days post-dose.

Exploratory pharmacokinetics (PK)/pharmacodynamics and biomarker endpoints included: MEDI3902 concentration and PK parameters in tracheal secretions through 21 days post-dose; cellular and protein markers of inflammation (WBC count and differential blood count in blood and procalcitonin and C-reactive protein [CRP] in serum) at baseline through 7 days post-dose in all subjects and through 49 days post-dose in subjects with suspected or documented serious *P. aeruginosa* infections; *ex vivo* anti-cytotoxicity and opsonophagocytic activity before and after administration of MEDI3902.

Specimen collection and schedule of study procedures for exploratory endpoints

Tracheal aspirate samples were collected to assess MEDI3902 PK in the lower respiratory tract from intubated subjects pre-dose on day 1 and on days 2, 4, 8, 15, and 22 of follow-up and the day of extubation if this occurred between scheduled assessments. Tracheal aspirate samples to assess for *P. aeruginosa* colonisation were collected within 36 hours before randomisation. Tracheal aspirate samples were collected to assess *P. aeruginosa* colonisation status by PCR on days 4, 8, 15, and 22, provided the subject remained intubated. While the subject was intubated, tracheal aspirate was also assessed for colonisation by PCR on day 1 of infection in subjects with suspected or confirmed pneumonia or bacteraemia and on day 4 of infection in subjects who were positive for *P. aeruginosa* bacteraemia or *P. aeruginosa* pneumonia. Blood samples for culture were taken pre-dose on day 1, and tracheal aspirate samples for culture were taken on days 2 and 4. Blood and serum for cellular and protein markers of inflammation were collected in all subjects pre-dose on day 1 and on day 8, in subjects with suspected or confirmed *P. aeruginosa* infections on day 1 of infection, and in those who

were positive for *P. aeruginosa* bacteraemia or pneumonia on day 4 and day 5 post-infection through clinical resolution. Serum *ex vivo* anti-cytotoxicity and opsonophagocytic activity were assessed for all subjects before administration of MEDI3902 on day 1 and on days 2, 8, 15, 22, and 29, for subjects with suspected or confirmed pneumonia or bacteraemia on day 1 of infection, and for those who were positive for *P. aeruginosa* bacteraemia or pneumonia on day 4 and day 5 post-infection through clinical resolution.

Supplementary Results

Exploratory efficacy analyses

The incidence of positive PA colonisation through 21 days post-dose was numerically lower in the MEDI3902 1500 mg group (86·5% [32/37]) compared with that of the placebo group (91·8% [45/49]). The incidence of EAC-determined all-cause pneumonia through 21 days post-dose was numerically higher in the MEDI3902 1500 mg group (29·4% [25/85]) compared with the placebo group (20·5% [17/83]), with a corresponding RRR of –43·6% (80% CI –104·0%, –1·1%). Very few subjects had EAC-determined PA tracheobronchitis (1500 mg n=4; placebo n=1), with an RRR for MEDI3902 1500 mg of –290·6% (80% CI –1514·5%, 5·5%) compared to placebo. The incidence of EAC-determined *P. aeruginosa* pneumonia through 28 days post-dose was similar between the MEDI3902 1500 mg group (23·5% [20/85]) and the placebo group (22·9% [19/83]), with a slight risk increase for MED3902 1500 mg versus placebo (RRR of –2·8% [80% CI –47·3%, 28·3%]).

In subjects who had at least one serious PA infection, a single dose of MEDI3902 1500 mg resulted in an RRR of –13·9% (80% CI –53·3%, 15·3%) compared to placebo. The incidence of serious PA disease through 21 days post-dose by event attributable to PA is shown in supplementary table 3.

Healthcare resource utilisation through 21 days post-dose for subjects with and without EAC-determined PA pneumonia is shown in supplementary table 4.

Severity scores related to first EAC-determined PA pneumonia through 21 days post-dose are shown in supplementary table 8.

Mortality rates through 28 days post-onset of breakthrough nosocomial pneumonia caused by *P. aeruginosa* were higher in the MEDI3902 1500 mg group (47·4% [9/19]) compared with those of the placebo group (33·3% [5/15]). The incidence of all-cause mortality through 49 days post-dose was numerically higher in the MEDI3902 1500 mg group (28·2% [24/85]) compared with that of the placebo group (22·9% [19/83]). A single dose of MED3902 1500 mg resulted in a risk increase versus placebo for all-cause mortality through 49 days post-dose (RRR of –23·3% [80% CI –73·3%, 12·2%]).

In the post-hoc analysis, the RRR (80% CI) in all-cause mortality for MEDI3902 1500 mg versus placebo of 56·5% (11·9, 79·4%) among subjects with ANC $\leq 8{\cdot}17\times10^3$ cells/µL was greater than that observed in the overall population (p=0·11; mortality of 11·1% for MEDI3902 [n=4/36], versus 25·5% [n=12/47] for placebo). A post-hoc analysis adjusted for imbalance in baseline covariates resulted in an RRR of –20·8% (80% CI: –81·1%, 19·4%; p=0·55), and a sensitivity analysis examining the effect of study centre as an additional variable resulted in an RRR of –20.4% (80% CI: –80.6%, 19.8%; p=0·56).

Exploratory biomarker analyses

Cellular and protein markers of inflammation

There were trends towards larger changes from baseline to day 8 in the concentrations of procalcitonin and CRP in the MEDI3902 1500 mg group compared with the placebo group. In contrast, WBC and differential blood counts remained largely unchanged (supplementary table 9).

EVADE MEDI3902

Change from baseline in cellular and protein markers of inflammation through day of clinical resolution for subjects with EAC-determined PA is shown in supplementary table 10.

Anti-cytotoxicity and opsonophagocytic activity

A positive correlation was observed between *ex vivo* anti-cytotoxicity and serum concentrations of MEDI3902 1500 mg (supplementary figure 4a; R-square=0·61). Similarly, opsonophagocytic killing activity was positively correlated with serum concentrations of MEDI3902 (supplementary figure 4b; R-square=0·64).

Supplementary table 1: Number of randomised subjects by country

**Country (# of sites)**	**MEDI3902 500 mg N=16**	**MEDI3902 1500 mg N=87**	**Placebo N=85**
Austria (2)	0	0	2
Belgium (5)	4	12	12
Croatia (1)	0	1	3
Czech Republic (3)	0	2	1
France (13)	6	34	34
Greece (5)	3	8	8
Hungary (2)	1	3	2
Israel (1)	0	4	4
Portugal (1)	0	1	1
Spain (8)	2	13	10
Turkey (3)	0	5	4
United Kingdom (2)	0	2	3
United States (2)	0	2	1

Supplementary table 2: List of participating principal investigators by country and site

**Country**	**Investigator name**	**Hospital**
Austria	Michael Joannidis	Medical University of Innsbruck
Austria	Walter Klimscha	SMZOST Donauspital Wien
Belgium	Elisabeth De Waele	UZ Brussel
Belgium	Nicolas De Schryver	Clinique Saint-Pierre
Belgium	Jacques Devriendt	CHU Brugmann
Belgium	Vincent Huberlant	Centre Hospitalier Jolimont-Lobbes
Belgium	Pieter Depuydt	University Hospital Gent
Belgium	Marc Bourgeois	AZ Sint-Jan AV
Belgium	Sam Van Boxstael	Ziekenhuis Oost-Limburg
Croatia	Mladen Peric	Klinichki Bolnicki Centar Zagreb
Croatia	Jasminka Kopic	General Hospital Dr Josip Bencevic
Czech Republic	Michal Hanauer	Krajska zdravotni, a.s. – Nemocnice Decin, o.z.
Czech Republic	Tomas Hruby	Krajska zdravotni, a.s. – Nemocnice Teplice, o.z.
Czech Republic	Vladimir Sramek	Fakultni nemocnice u sv. Anny v Brne
Czech Republic	Petr Svoboda	Nemocnice Kyjov, prispevkova organizace
Czech Republic	Tomas Vymazal	Fakultni nemocnice v Motole
Czech Republic	Martin Novacek	Oblastni nemocnice Kolin, a.s.
France	Bruno François	Centre Hospitalier et Universitaire de Limoges
France	Djillali Annane	APHP Raymond-Poincaré de Garches
France	Jean Chastre	Groupe Hospitalier Pitié Salpétrière
France	Jean-Paul Mira	APHP Cochin
France	Bertrand Souweine	Centre Hospitalier Universitaire de Clermont Ferrand
France	Pierre-François Dequin	CHRU de Tours
France	Ferhat Meziani	Nouvel Hôpital Civil Strasbourg
France	François Stephan	Centre Chirurgical Marie Lannelongue
France	Saadalla Nseir	CHRU Lille
France	Sebastien Gibot	CHRU Nancy
France	Carole Schwebel	Hôpital Albert Michallon La Tronche
France	Alain Lepape	Centre Hospitalier Lyon Sud
France	Gaetan Plantefeve	Centre Hospitalier Victor Dupouy
France	Jean-Luc Diehl	APHP Hôpital Européen Georges-Pompidou
France	Christian Richard	APHP Hôpital de Bicêtre
France	Christian Lamer	Institut Mutualiste Montsouris
France	Kada Klouche	Centre Hospitalier Universitaire de Montpellier/ Lapeyronie hospital
France	Samir Jaber	Centre Hospitalier Universitaire de Montpellier / Hôpital St Eloi
Greece	Epaminondas Zakynthinos	University Hospital of Larissa
Greece	Georgios Filntisis	Agioi Anargyroi Cancer Hospital
Greece	Apostolos Komnos	General Hospital of Larissa
Greece	Spyros Zakynthinos	Evangelismos General Hospital of Athens

Greece	Antonia Koutsoukou	Sotiria Chest Hospital of Athens
Greece	Georgios Saroglou	Metropolitan Hospital
Greece	Charikleia Nikolaou	Konstantopouleion General Hospital of Athens
Greece	Glykeria Vlachogianni	Agios Dimitrios General Hospital of Thessaloniki
Greece	Ioannis Pnevmatikos	University Hospital of Alexandroupolis
Greece	Konstantinos Mandragos	General Hospital of Athens Korgialenio Benakio Greek Red Cross
Hungary	Ildiko Kremer	Pest Megyei Flór Ferenc Kórház
Hungary	Zsolt Dezso Rozgonyi	Orszagos Koranyi TBC es Pulmonologiai Intezet
Hungary	Zsuzsa Marjanek	Jávorszky Ödön Kórház
Ireland	Ignacio Martin-Loeches	St James University Hospital
Israel	Pierre Singer	Rabin Medical Center
Israel	Vernon Van Heerden	Hadassah Medical Center
Israel	Yehuda Carmeli	Tel Aviv Sourasky Medical Center
Israel	Galia Rahav	Chaim Sheba Medical Center
Portugal	Pedro Povoa	Centro Hospital de Lisboa Ocidental – Hospital São Francisco Xavier
Portugal	Antonio Alvarez Seoane	Centro Hospitalar Lisboa Norte, E.P.E. – Hospital de Santa Maria
Portugal	Pedro Moura	Unidade Local de Saúde do Alto Minho, EPE
Portugal	Filipe Gonzalez	Hospital Garcia de Orta
Spain	Paula Ramirez	Hospital Universitari i Politecnic La Fe de Valencia
Spain	Antonio Torres Marti	Hospital Clinic de Barcelona
Spain	Miguel Sánchez-García	Hospital Clínico San Carlos Madrid
Spain	Ricard Ferrer Roca	Hospital Universitario Vall d'Hebron Barcelona
Spain	Lorena Oteiza	Hospital Universitario de Getafe Madrid
Spain	Dolores Escudero	Hospital Universitario Central de Asturias Oviedo
Spain	Enrique Piacentini	Hospital Mutua de Terrassa Barcelona
Spain	Paula Vera	Hospital de La Santa Creu i Sant Pau
Spain	Luis Tamayo	Hospital Universitario del Rio Hortega
Spain	Miguel Angel Gonzalez Gallego	Hospital Universitario Infanta Sofia
Spain	Borja Suberviola Canas	Hospital Universitario Marqués de Valdecilla
Spain	Iglesias Figueira	Hospital Universitario La Paz – PPDS
Spain	Rafael Leon	Hospital General Universitario Reina Sofia
Turkey	Volkan Korten	Marmara University Research and Training Hospital
Turkey	Iftihar Koksal	Karadeniz Technical University Faculty of Medicine
Turkey	Murat Akova	Hacettepe Universitesi Tip Fakultesi Hastanesi
United Kingdom	Duncan Wyncoll	St Thomas' Hospital
United Kingdom	Tony Whitehouse	Queen Elizabeth Hospital
United Kingdom	Phil Hopkins	King's College Hospital
United Kingdom	Malcolm Sim	Southern General Hospital
United States	Yoav Golan	Tufts University Medical Center
United States	Marcus Zervos	Henry Ford Health Sys. Detroit
United States	Jose Vazquez	Georgia Regents Medical Center-Augusta

United States	Kartikeya Cherabuddi	University of Florida
United States	George Smulian	Univ. of Cincinnati
United States	Nadine Rouphael	Emory University Atlanta
United States	James Welker	Anne Arundel Health
United States	Mathew Sims	Beaumont Hospital Royal Oaks
United States	David Van Duin	UNC Chapel Hill
United States	Todd McCarthy	Univ. of Alabama, Birmingham
United States	Christopher Polk	Carolina Medical Center/Atrium Health

Supplementary table 3: Ventilator-associated pneumonia prevention measures used in placebo and MEDI3902 patients (mITT)

**VAP prevention measures**	**MEDI3902 500 mg n = 16**	**MEDI3902 1500 mg n = 85**	**Placebo n = 83**	**MEDI3902 Total n = 101**	**Total n = 184**
Elevation of the head of the bed					
Yes	14 (87.5%)	82 (96.5%)	82 (98.8%)	96 (95.0%)	178 (96.7%)
No	2 (12.5%)	3 (3.5%)	1 (1.2%)	5 (5.0%)	6 (3.3%)
Missing	0	0	0	0	0
Daily sedation vacations and assessment of readiness to extubate					
Yes	16 (100%)	57 (67.1%)	56 (67.5%)	73 (72.3%)	129 (70.1%)
No	0	28 (32.9%)	27 (32.5%)	28 (27.7%)	55 (29.9%)
Missing	0	0	0	0	0
Peptic ulcer disease prophylaxis					
Yes	16 (100%)	73 (85.9%)	69 (83.1%)	89 (88.1%)	158 (85.9%)
No	0	12 (14.1%)	14 (16.9%)	12 (11.9%)	26 (14.1%)
Missing	0	0	0	0	0
Deep venous thrombosis prophylaxis					
Yes	15 (93.8%)	78 (91.8%)	77 (92.8%)	93 (92.1%)	170 (92.4%)
No	1 (6.3%)	7 (8.2%)	6 (7.2%)	8 (7.9%)	14 (7.6%)
Missing	0	0	0	0	0
Daily oral care with chlorhexidine					
Yes	11 (68.8%)	69 (81.2%)	61 (73.5%)	80 (79.2%)	141 (76.6%)
No	5 (31.3%)	16 (18.8%)	22 (26.5%)	21 (20.8%)	43 (23.4%)
Missing	0	0	0	0	0
All 5 measures used					
Yes	8 (50.0%)	36 (42.4%)	31 (37.3%)	44 (43.6%)	75 (40.8%)
No	8 (50.0%)	49 (57.6%)	52 (62.7%)	57 (56.4%)	109 (59.2%)
Missing	0	0	0	0	0

mITT = modified intent-to-treat population; VAP = ventilator-associated pneumonia

Supplementary table 4: Healthcare resource utilisation through 21 days post-dose for subjects with and without EAC-determined PA pneumonia

	**Duration (days) per 22 patient-days of follow-up for subjects without PA pneumonia**		**Duration (days) per 22 patient-days of follow-up for subjects with PA pneumonia**	
	**MEDI3902 1500 mg (n=66)**	**Placebo (n=68)**	**MEDI3902 1500 mg (n=19)**	**Placebo (n=15)**
Hospitalisation	21·3	21·0	21·7	22·0
ICU stay	17·6	18·2	20·6	21·7
Mechanical ventilation	13·1	15·1	17·5	19·7
Supplemental oxygen	6·5	5·4	5·0	5·5
Anti-PA antibiotic usage	3·1	3·0	1·2	1·4
Systemic antibiotic usage	7·5	10·5	12·3	14·4

EAC=Endpoint Adjudication Committee. PA=*Pseudomonas aeruginosa*. ICU=intensive care unit.

Duration per 22 patient-days of follow-up time = (total duration of healthcare resource utilisation measure/sum of follow-up time) × 22 days.

Supplementary table 5: Incidence (n, %) of serious PA disease through 21 days post-dose by event attributable to PA

	**MEDI3902 1500 mg (n=85)**	**Placebo (n=83)**
Tracheobronchitis	4 (4.7%)	1 (1.2%)
Bacteraemia	6 (7·1%)	4 (4·8%)
Deep skin or tissue infection*	0	3 (3·6%)
Intra-abdominal infection*	1 (1·2%)	2 (2·4%)
Meningitis*	1 (1·2%)	0

PA=*Pseudomonas aeruginosa*.

*Serious PA infections obtained through medical monitor review of adverse events.

Supplementary table 6: Subjects with PA pneumonia (not EAC-determined) with anti-PA effective antibiotics, which overlap the adverse event as-treated population

	Placebo<br>n = 83	MEDI3902<br>500mg<br>n = 16	MEDI3902<br>1500 mg<br>n = 85	MEDI3902<br>Total<br>n = 101
Infectious pleural effusion	0	0	1 (1.2%)	1 (1.0%)
Lung infection pseudomonal	0	0	1 (1.2%)	1 (1.0%)
Pneumonia bacterial	1 (1.2%)	1 (6.3%)	0	1 (1.0%)
Pneumonia pseudomonal	3 (3.2%)	0	0	0
Pseudomonas infection	0	0	1 (1.2%)	1 (1.0%)
Total	4 (4.8%)	1 (6.3%)	2 (2.4%)	3 (3.0%)

EAC=Endpoint Adjudication Committee. PA=*Pseudomonas aeruginosa*.

Subjects are counted only once regardless of the number of events

Supplementary tables 7A and 7B: PA pneumonia incidence by procalcitonin and absolute neutrophil count quartiles at baseline – (mITT)

Table 7A

Procalcitonin levels at baseline	MEDI3902 1500mg	Placebo	Relative Risk Reduction	80% Confidence Interval
Quartile 1: ≤0.1 µg/L	2/21 (9.5%)	2/20 (10.0%)	4.80%	-238.7% to 72.2%
Quartile 2: >0.1 - ≤0.2 µg/L	2/18 (11.1%)	4/17 (23.5%)	52.80%	-40.0% to 83.1%
Quartile 3: >0.2 - ≤0.55 µg/L	3/17 (17.6%)	8/22 (36.4%)	51.50%	-4.4% to 79.8%
Quartile 4: > 0.55 µg/L	9/21 (42.9%)	0/15 (0.0%)	NA	NA
Procalcitonin level: ≤0.55 µg/L	7/56 (12.5%)	14/59 (23.7%)	47.3%	6.1% to 69.9%

Table 7B

ANC count at Baseline	MEDI3902 1500mg	Placebo	Relative Risk Reduction	80% Confidence Interval
Quartile 1: ≤5.71×10^3/µL	1/19 (5.3%)	2/22 (9.1%)	42.10%	-155.7% to 87.6%
Quartile 2: >5.71 - ≤8.17×10^3/µL	0/17 (0.0%)	6/25 (24.0%)	100.00%	52.9% to 100.0%
Quartile 3: >8.17 - ≤12.6×10^3/µL	6/20 (30.0%)	4/21 (19.0%)	-57.50%	-251.8% to 26.3%
Quartile 4: > 12.6×10^3/µL	10/27 (37.0%)	3/13 (23.1%)	-60.50%	-284.0% to 21.8%
ANC count: ≤8.17×10^3/µL	1/36 (2.8%)	8/47 (17.0%)	83.7%	39.5% to 95.5%

Supplementary table 8: Severity scores (mean [standard deviation]) related to first EAC-determined PA pneumonia through 21 days post-dose in subjects with EAC-determined PA pneumonia

	**MEDI3902 1500 mg (n=19)**	**Placebo (n=15)**
CPIS		
Baseline*	3·7 (1·3)	3·2 (1·3)
Day 1 of onset	6·5 (1·3)	5·8 (2·2)
Day of resolution	4·3 (1·7)	3·5 (2·3)
SOFA		
Baseline*	5·9 (2·6)	4·5 (1·9)
Day 1 of onset	8·8 (3·7)	5·5 (3·2)
Day of resolution	5·4 (2·8)	3·4 (2·8)

CPIS=Clinical Pulmonary Infection Score. EAC=Endpoint Adjudication Committee. PA=*Pseudomonas aeruginosa*. SOFA=Sequential Organ Failure Assessment.

*Baseline indicates last assessment before the dose.

Supplementary table 9: Change from baseline (mean [standard deviation]) in cellular and protein markers of inflammation through day 8 (all subjects, mITT)

	**MEDI3902 1500 mg (n=85)**	**Placebo (n=83)**
WBC, 10^3 cells/µL	–0·30 (6·74)	–0·13 (3·95)
Neutrophils, 10^3 cells/µL	–0·12 (6·55)	0·03 (4·10)
Lymphocytes, 10^3 cells/µL*	0·03 (0·66)	0·10 (0·78)
Monocytes, 10^3 cells/µL†	–0·01 (0·39)	0·06 (0·48)
Eosinophils, 10^3 cells/µL‡	0·03 (0·21)	0·02 (0·23)
Basophils, 10^3 cells/µL§	–0·01 (0·07)	0·00 (0·08)
Serum procalcitonin, µg/L	1·65 (13·74)	0·13 (1·70)
Serum CRP, mg/dL	–3·76 (16·94)	–1·49 (19·39)

CRP=c-reactive protein. mITT=modified intent-to-treat. SD=standard deviation. WBC=white blood cell.

*Baseline values were 1·30 (0·72) $\times 10^3$ cells/µL for MEDI3902 1500 mg and 1·37 (0·82) $\times 10^3$ cells/µL for placebo; †baseline values were 0·82 (0·48) $\times 10^3$ cells/µL for MEDI3902 1500 mg and 0·76 (0·35) $\times 10^3$ cells/µL for placebo; ‡baseline values were 0·22 (0·29) $\times 10^3$ cells/µL for MEDI3902 1500 mg and 0·27 (0·28) $\times 10^3$ cells/µL for placebo; §baseline values were 0·05 (0·06) $\times 10^3$ cells/µL for MEDI3902 1500 mg and 0·05 (0·06) $\times 10^3$ cells/µL for placebo.

For baseline values for WBC, neutrophils, procalcitonin, and CRP, please see table 1.

Supplementary table 10: Change from baseline in cellular and protein markers of inflammation through day of clinical resolution for subjects with EAC-determined PA pneumonia* (mITT)

	**MEDI3902 1500 mg (n=19)**	**Placebo (n=15)**
Mean (SD) WBC, 10^3 cells/µL*,†		
Day 1 of onset	1·84 (6·88)	1·45 (5·98)
Day 4 of onset	–0·69 (7·94)	–0·11 (2·53)
Day of resolution	–2·67 (5·75)	0·22 (3·58)
Mean (SD) neutrophils, 10^3 cells/µL‡		
Day 1 of onset	0·64 (4·46)	1·77 (6·68)
Day 4 of onset	0·67 (7·37)	0·26 (2·66)
Day of resolution	–2·50 (5·37)	–0·46 (4·15)
Mean (SD) lymphocytes, 10^3 cells/µL§		
Day 1 of onset	0·07 (0·57)	–0·08 (0·44)
Day 4 of onset	0·003 (0·64)	0·31 (0·47)
Day of resolution	0·23 (0·54)	0·56 (0·54)
Mean (SD) monocytes, 10^3 cells/µL¶		
Day 1 of onset	0·16 (0·52)	0·02 (0·41)
Day 4 of onset	0·12 (0·32)	–0·02 (0·29)
Day of resolution	0·04 (0·40)	0·01 (0·42)
Mean (SD) eosinophils, 10^3 cells/µL#		
Day 1 of onset	0·03 (0·46)	–0·06 (0·16)
Day 4 of onset	0·15 (0·36)	–0·03 (0·13)
Day of resolution	0·17 (0·53)	–0·02 (0·16)
Mean (SD) basophils, 10^3 cells/µL**		
Day 1 of onset	–0·02 (0·08)	–0·01 (0·03)
Day 4 of onset	–0·004 (0·07)	0·04 (0·05)

Day of resolution	–0·01 (0·08)	0·01 (0·03)
Mean (SD) serum procalcitonin, µg/L††		
Day 1 of onset	–0·03 (4·31)	0·42 (0·61)
Day 4 of onset	–0·37 (1·27)	0·46 (0·90)
Day of resolution	–1·42 (4·44)	0·08 (0·19)
Mean (SD) serum CRP, mg/dL‡‡		
Day 1 of onset	4·36 (5·88)	9·01 (10·10)
Day 4 of onset	–1·21 (6·87)	5·27 (11·17)
Day of resolution	–3·12 (2·98)	–4·04 (6·32)

CRP=c-reactive protein. mITT=modified intent-to-treat. SD=standard deviation. WBC=white blood cell.

*Includes data collected through day 49 for subjects with EAC-determined *P. aeruginosa* pneumonia with onset through day 22; †baseline values were 16·49 (5·38) $\times10^3$ cells/µL for MEDI3902 1500 mg and 13·63 (10·32) $\times10^3$ cells/µL for placebo; ‡baseline values were 14·16 (5·39) $\times10^3$ cells/µL for MEDI3902 1500 mg and 9·35 (5·09) $\times10^3$ cells/µL for placebo; §baseline values were 1·07 (0·67) $\times10^3$ cells/µL for MEDI3902 1500 mg and 0·99 (0·52) $\times10^3$ cells/µL for placebo; ¶baseline values were 0·76 (0·41) $\times10^3$ cells/µL for MEDI3902 1500 mg and 0·66 (0·33) $\times10^3$ cells/µL for placebo; #baseline values were 0·26 (0·26) $\times10^3$ cells/µL for MEDI3902 1500 mg and 0·24 (0·34) $\times10^3$ cells/µL for placebo; **baseline values were 0·06 (0·06) $\times10^3$ cells/µL for MEDI3902 1500 mg and 0·04 (0·04) $\times10^3$ cells/µL for placebo; ††baseline values were 2·16 (3·49) µg/L for MEDI3902 1500 mg and 0·26 (0·16) µg/L placebo; ‡‡baseline values were 10·71 (7·31) mg/dL for MEDI3902 1500 mg and 9·34 (6·99) mg/dL for placebo.

Supplementary table 11: Subgroups by Baseline Disease (mITT)

**Characteristics**		**Interaction p-value**[a]	**MEDI3902 1500mg (n=85)**	**MEDI3902 500mg (n=16)**	**Placebo (n=83)**	**RRR**[b]	**80% CI**[b]
Severe COPD[c]	Yes	0.643	2/4 (50.0%)	0/1	¼ (25.0%)	-100.0%	(-544.3%, 47.8%)
	No		17/81 (21.0%)	2/15 (13.3%)	13/78 (16.7%)	-25.9%	(-95.7%, 19.8%)
Antibiotics usage	Yes	0.083	15/59 (25.4%)	2/14 (14.3%)	9/63 (14.3%)	-78.0%	(-195.4%, 4.9%)
	No		2/20 (10.0%)	0/1	4/14 (28.6%)	65.0%	(-4.4%, 88.2%)
Previous PA infections	Yes	0.015	11/25 (44.0%)	1/7 (14.3%)	4/31 (12.9%)	-241.0%	(-594.2%, -70.6%)
	No		8/60 (13.3%)	1/9 (11.1%)	10/51 (19.6%)	32.0%	(-22.1%, 62.0%)

[a] the interaction p-value is obtained from Poisson regression with robust variance, including terms of treatment group, subgroup being tested, and treatment by subgroup interaction.

[b] Relative risk reduction (MEDI3902 1500mg versus placebo) and 80% confidence interval (CI) based on unconditional confidence interval on ratio proportions.

[c] Severe COPD = Severe COPD Global Initiative for Chronic Obstructive Lung Disease (GOLD) III or IV

COPD = Chronic Obstructive Pulmonary Disease, mITT=modified intent-to-treat.

Supplementary figure 1: Study design

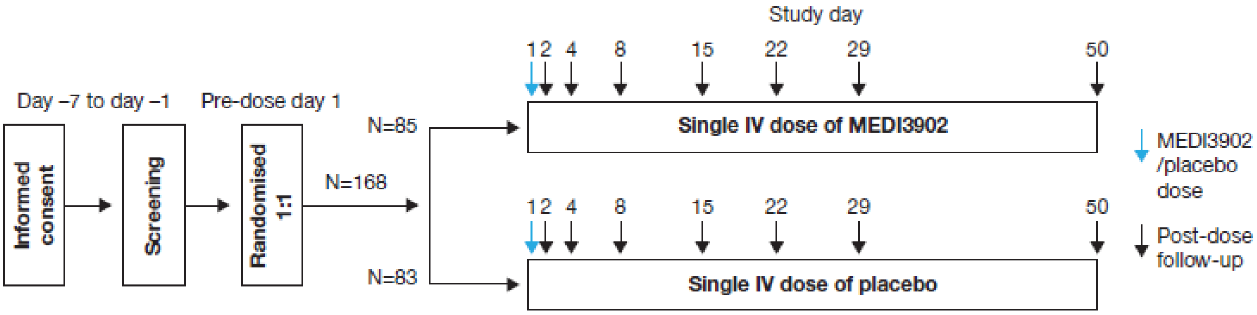

Patients were randomised following positive PCR to identify *P. aeruginosa* colonisation in the lower respiratory tract.

IV=intravenous. PCR=polymerase chain reaction.

Supplementary figure 2. Cumulative incidence function for EAC-determined PA pneumonia with death as a competing risk (mITT)

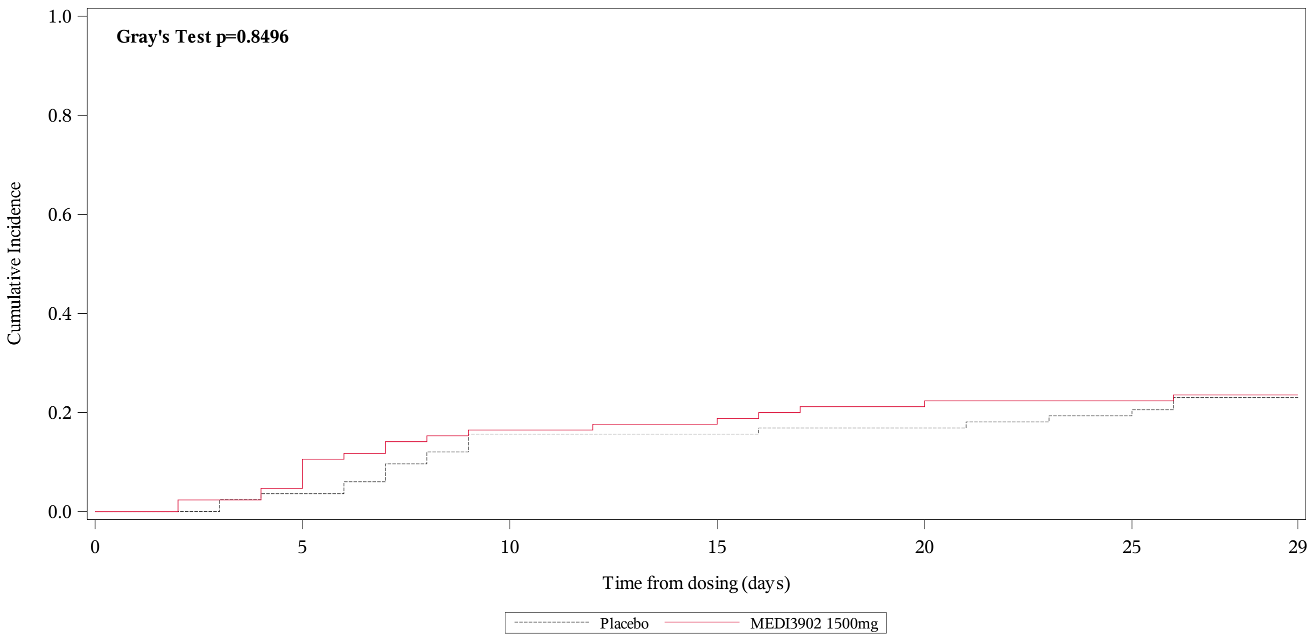

EAC= Endpoint Adjudication Committee. mITT =modified intention-to-treat population

Supplementary figure 3: Impact of baseline covariates on MEDI3902 efficacy against PA pneumonia in the overall study (post-hoc analysis, mITT)

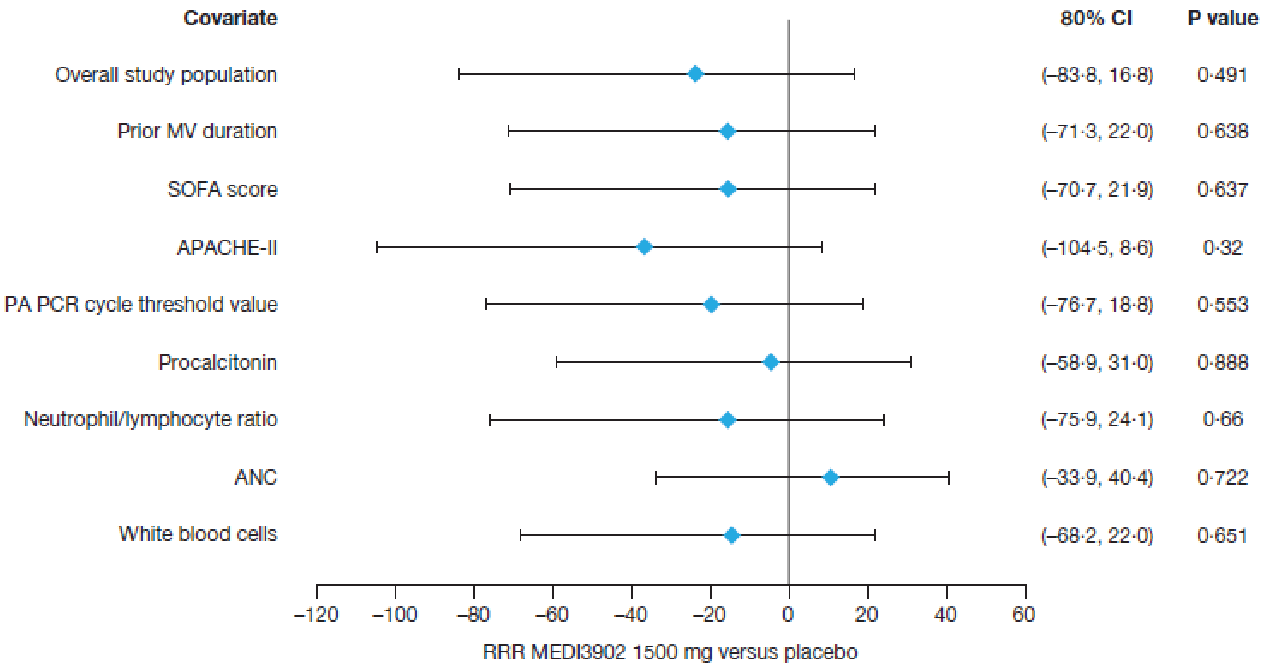

RRR MEDI3902 1500 mg versus placebo and 80% CI. ANC=absolute neutrophil count. APACHE-II=Acute Physiology and Chronic Health Evaluation-II. CI=confidence interval. MV=mechanical ventilation. mITT=modified intent-to-treat population. PCR=polymerase chain reaction. RRR=relative risk reduction. SOFA=Sequential Organ Failure Assessment.

Supplementary figure 4: Correlation between anti-cytotoxicity (a) or opsonophagocytic killing activity (b) and serum concentrations of MEDI3902 following 1500 mg dosing

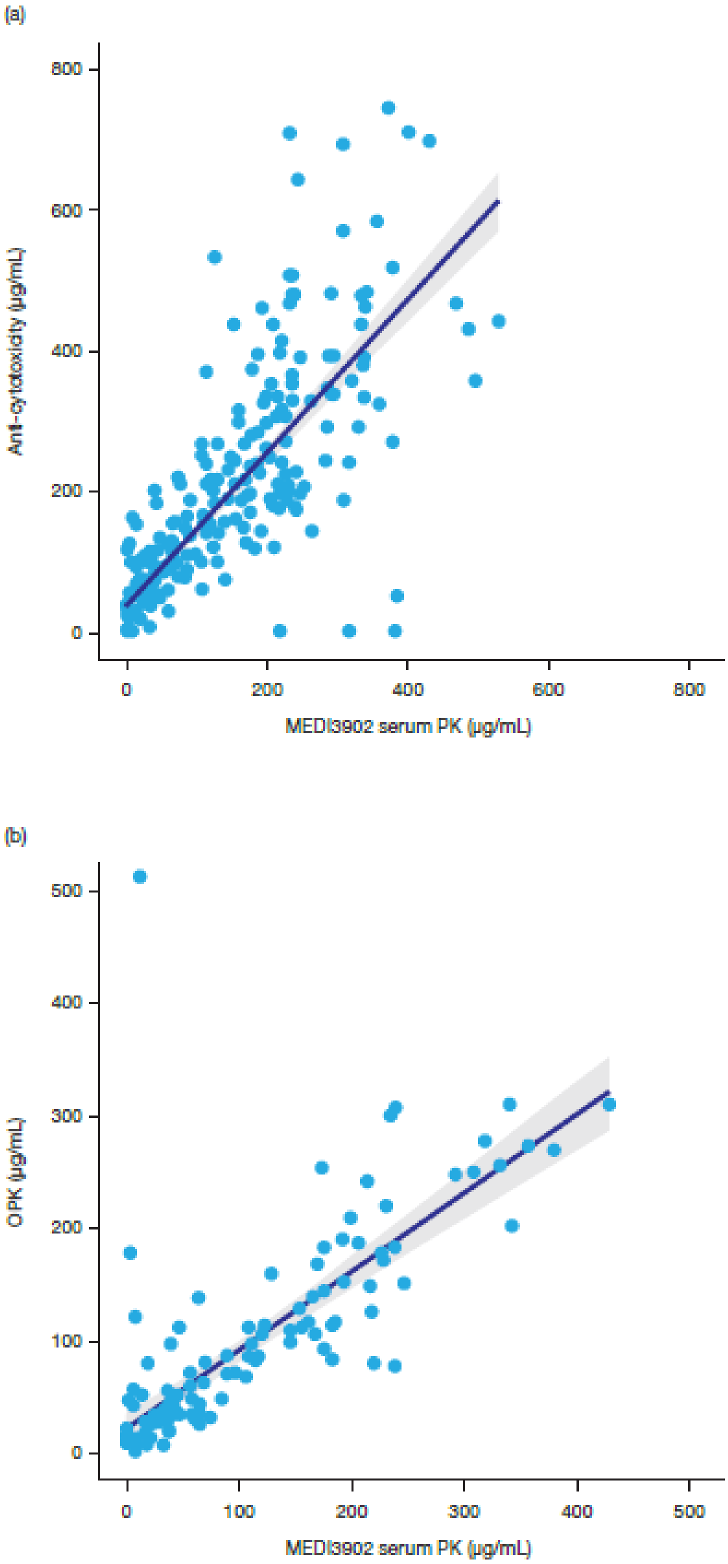

OPK=opsonophagocytic killing activity. PK=pharmacokinetics.

Supplementary figure 5: MEDI3902 1500 mg endotracheal aspirate pharmacokinetic (PK) by visit.

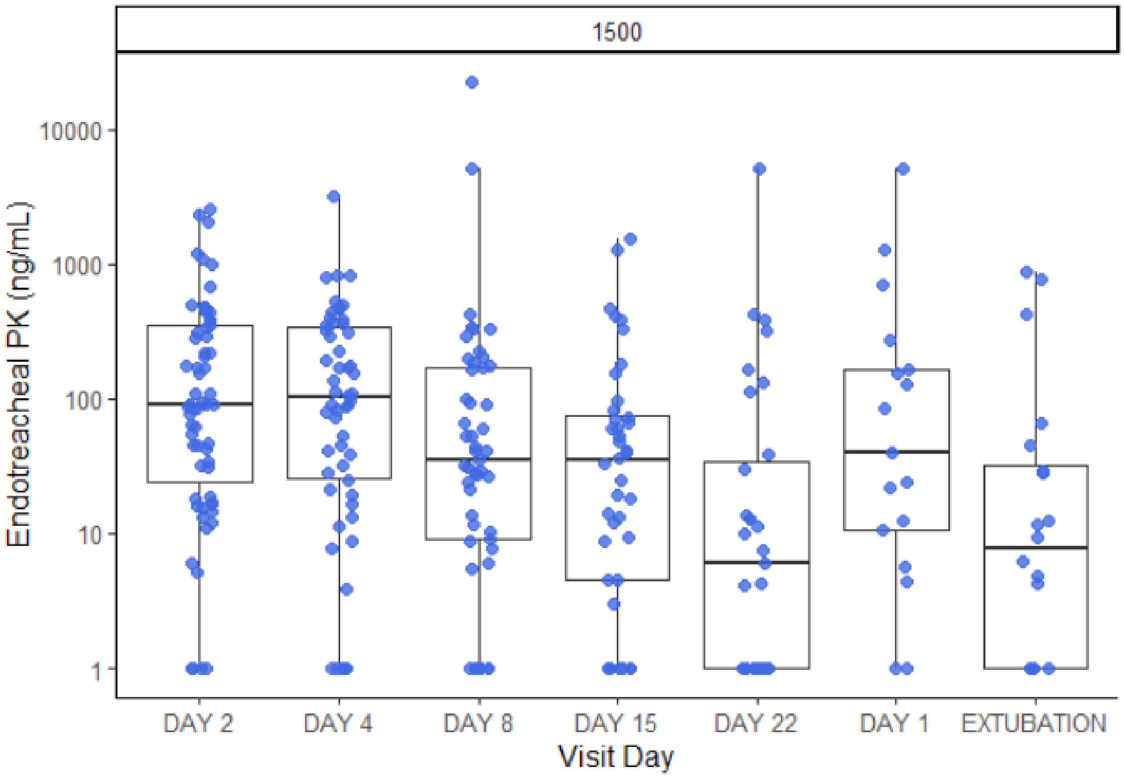

Supplementary figure 6: Exposure–response relationship between MEDI3902 1500 mg AUC0–21 and the likelihood of developing PA pneumonia

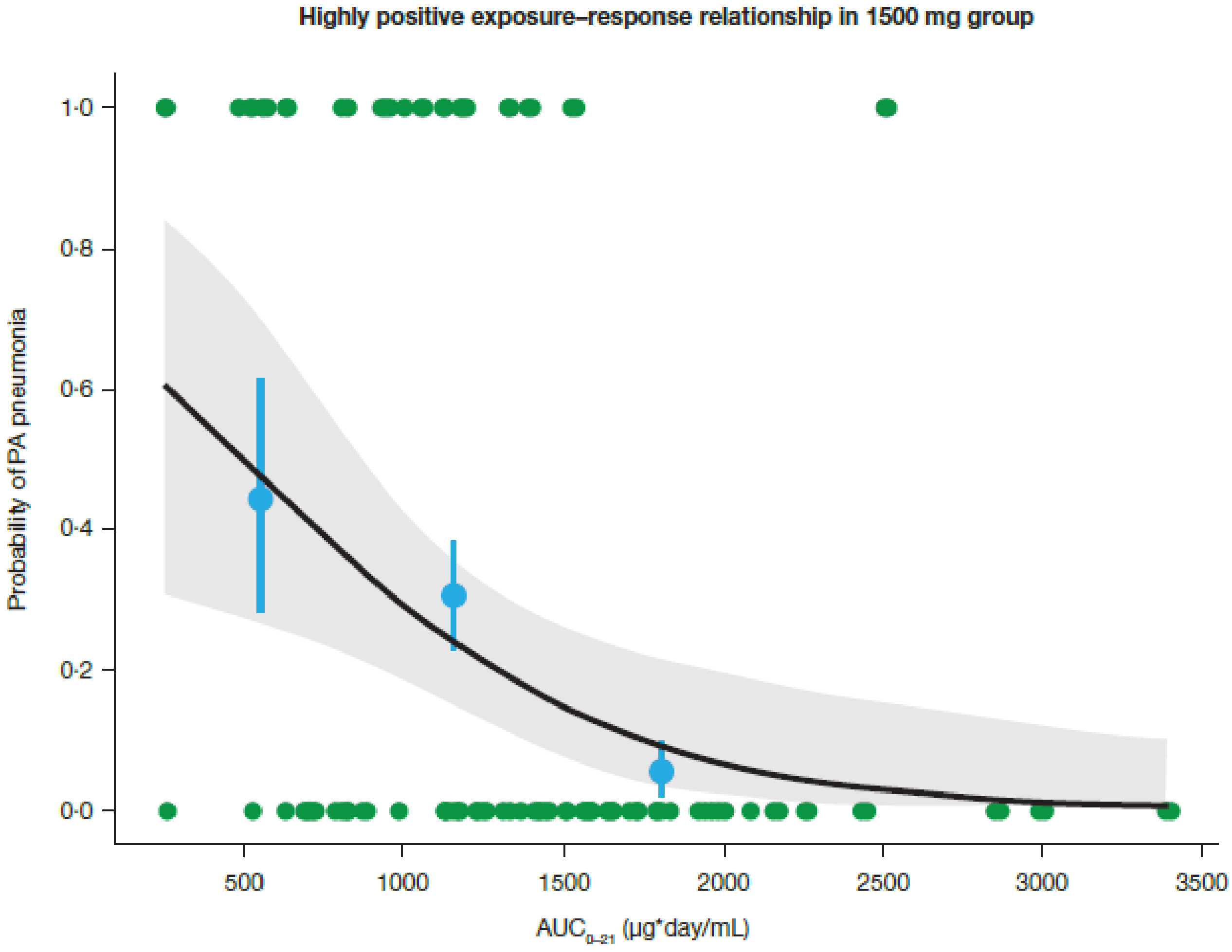

Solid black line: the median-predicted probability of PA pneumonia. Grey shades: 90% confidence interval of the predicted probability of PA pneumonia. Green dots: observed data with y=0 indicating no PA pneumonia within 21 days and y=1 indicating PA pneumonia onset within 21 days. Blue dots and error bars: observed proportion (±SE) of subjects with PA pneumonia by tertiles of AUC_{0-21}.

AUC_{0-21}=the area under the concentration–time curve from time zero to 21 days post-dose. PA=*P. aeruginosa*. SE=standard error.
